# Comparative Analysis of Test Methods for Predicting Dermal Sensitization Potency in Essential Oils: A Component-Based Prediction Model, GARDskin Dose–Response Assay, and Local Lymph Node Assay

**DOI:** 10.3390/toxics14090800

**Published:** 2026-09-09

**Authors:** Joseph T. Dawson, Tim Lindberg, Prabodh Satyal, Ambika Poudel, Dakota T. Carter, Sara A. Shah, Cécile Bascoul

**Affiliations:** 1 dōTERRA International, Pleasant Grove, UT 84062, USA; psatyal@doterra.com (P.S.); apoudel@doterra.com (A.P.); dtcarter@doterra.com (D.T.C.); sshah@doterra.com (S.A.S.); cbascoul@doterra.com (C.B.); 2 Senzagen AB, 22381 Lund, Sweden; tim.lindberg@senzagen.com

**Keywords:** skin sensitization, risk assessment, essential oils, mixtures, non-animal methods, new approach methodologies (NAMs), component-based prediction, GARDskin dose–response, local lymph node assay, comparative analysis

## Abstract

The dose–response adaptation of the genomic allergen rapid detection for skin sensitizers (GARDskin DR) assay is a non-animal method for predicting sensitization potency. This proof-of-concept comparative analysis evaluates outputs from a component-based prediction model (CP), GARDskin DR, and historical local lymph node assays (LLNA), exploring the applicability of CP and GARDskin DR for evaluating the no-expected-sensitization induction level (NESIL) potencies of essential oils. Eight well-studied essential oils were selected: cedarwood, cinnamon bark, clove bud, geranium, lavender, lemongrass, spearmint, and tea tree oils. Each underwent GARDskin DR as well as GC–MS, from which CP model outputs were derived. GARDskin DR, CP, and LLNA outputs were compared graphically and using preliminary descriptive statistics. Results show overall statistical concordance in material ranking between methods (Kendall’s W = 0.857, *p* = 0.017). Bland–Altman log-transformed bias and limits of agreement show numerically higher NESIL_CP_ compared to corresponding NESIL_GARD_ and NESIL_LLNA_, but sensitivity analysis shows this finding is statistically sensitive to geranium oil. Spearman’s rank correlations were significant between NESIL_CP_ vs. NESIL_GARD_ and NESIL_LLNA_ but not between NESIL_GARD_ vs. NESIL_LLNA_ despite numerical similarity. Given the small sample, no statistical findings from this study are generalizable and should be interpreted descriptively. However, the NESIL results provide preliminary evidence supporting further evaluation of GARDskin DR and CP for dermal sensitization potency testing of essential oils.

## 1. Introduction

The Global Harmonized System of Classification and Labeling of Chemicals (GHS) provides standards for categorizing and communicating the hazards of dermally sensitizing substances [1]. These guidelines classify dermal sensitizers as substances that induce an allergic response following skin contact. Reactions may occur after acute, repeated, and/or delayed exposure to dermal sensitizers and include allergy induction and allergy elicitation reactions. Data to classify dermal sensitizers come from human, animal, and in vitro research. Mixtures, in the absence of data on the complete or a similar material, are classified as dermal sensitizers if they contain ≥0.1% of a skin sensitizer category 1A constituent or ≥1.0% of a skin sensitizer category 1B constituent. All dermally sensitizing substances are labeled H317 skin sensitizers with the warning “may cause allergic skin reaction.”

When a GHS hazard is identified, a risk assessment is performed to model the likelihood of that hazard occurring in a practical setting. Dermal sensitization risk is based on the dermal sensitizing potency of a material and the end user’s exposure [2,3]. If the material is a mixture, then compositional information about the material subcomponents and their dermal sensitization potency are often used to estimate risk [4]. In doing so, risk assessors assume that a single constituent or constituent group drives the dermal sensitization effect of a mixture based on the observation that cross-reactivity between sensitizing compounds is rare [5].

Historically, dermal sensitization risk assessments were based on results from the murine LLNA, which characterizes a material’s dermal sensitization dose–response effect and determines a point of departure (POD) known as the NESIL [6]. NESIL values are used to inform safe starting exposures for human repeat insult patch tests (HRIPT), and a low-risk exposure threshold is confirmed clinically using the confirmation of no induction in humans (CNIH) protocol [7]. While the LLNA is regarded as a refined method within the 3R principles to replace, refine, and reduce the use of animals in research, new approach methodologies (NAMs) [8], such as the skin allergy risk assessment-integrated chemical environment (SARA-ICE) and GARDskin DR, use in vitro models that address technical limitations associated with the LLNA [9,10,11,12,13,14,15,16,17,18,19].

GARDskin DR is based on the GARDskin assay outlined in Organisation for Economic Co-operation and Development Test Guideline (OECD TG) 442E [20], which is included in the OECD TG 497 [21] “two out of three” defined approach as a method to test for key event 3 in the identification of dermal sensitization hazards. Studies have demonstrated that GARDskin DR-derived NESIL predictions for discrete compounds are generally consistent with values derived from human and LLNA data, supporting its potential utility for evaluating the dermal sensitizing potency of natural complex substances (NCSs) [12,13,22,23,24].

Many essential oils and other NCSs are classified as skin sensitizers under the GHS criteria due to the presence of dermally sensitizing constituents. While the dermally sensitizing constituents present in NCS materials may be clinically significant at high concentrations, the risk of dermal sensitization can be mitigated by dilution. Many essential oils are used in topical consumer products and are in increasing demand and availability for direct consumer use. Consequently, robust approaches for evaluating dermal sensitization risk are needed to support recommendations for safe use of essential oils and are especially important for developing new varieties and uses.

There is a small, yet growing body of dermal sensitization NAM research for NCSs [12,23,25,26,27,28,29]. This study adds to that body of research by comparing dermal sensitizing potency values for eight essential oil materials generated using three separate methods: GARDskin DR, LLNA, and a CP model that estimates a material’s NESIL based on the concentration and sensitizing potency of its discrete constituents. This approach provides insight into the applicability of GARDskin DR and CP for the eight essential oils evaluated.

## 2. Materials and Methods

### 2.1. Test Materials

Essential oils from cedarwood (CAS 8000-27-9), cinnamon bark (CAS 8007-80-5), clove bud (CAS 8000-34-8), geranium (CAS 8000-46-2), lavender (CAS 90063-37-9), lemongrass (CAS 8007-02-1), spearmint (CAS 8008-79-5), and tea tree (CAS 68647-73-4) were provided by dōTERRA, Pleasant Grove, UT, USA. These test materials were selected based on three criteria: (1) the availability of sufficient historical data to establish a dermal sensitization classification for the whole essential oil, (2) the reproducibility of the essential oil chemistry, and (3) sufficient data on the essential oil constituents to estimate a constituent-based dermal sensitization POD using the CP model. All test materials were authenticated by P.S. and A.P. Descriptions of the test materials are shown in Table 1. See Supplementary Materials Table S1 for manufacturing and test dates.

### 2.2. GC–MS Analysis

Essential oils were analyzed by GC–MS using a Shimadzu GCMS-QP2010 Ultra operated in electron ionization mode (electron energy = 70 eV), scan range = 40–400 atomic mass units, scan rate = 3.0 scans/s, and GCMSsolution software (version 4.52, Shimadzu Scientific Instruments, Columbia, MD, USA). The GC column was a ZB-5 fused silica capillary column with a (5% phenyl)-polymethylsiloxane stationary phase and a film thickness of 0.25 μm, a length of 30 m, and an internal diameter of 0.25 mm (Phenomenex, Torrance, CA, USA). The carrier gas was helium with a column head pressure of 552 kPa and flow rate of 1.37 mL/min. The injector temperature was 250 °C and the ion source temperature was 200 °C. The GC oven temperature was programmed for 50 °C initial temperature, then temperature was increased at a rate of 2 °C/min to 260 °C. A 7% *w*/*v* solution of the sample was prepared in dichloromethane and 0.1 μL was injected with a splitting mode (30:1). Identification of the oil components was based on their retention indices determined by reference to a homologous series of n-alkanes, and by comparison of their mass spectral fragmentation patterns with those reported in the literature [30] and an in-house library [31].

### 2.3. Component-Based Prediction Model

The component-based prediction utilized in this study is a deterministic model that estimates the dermal sensitization potency of a mixture based on the concentration and potency of a single-most driving constituent. The model calculates the dermal sensitization POD of a mixture (*NESIL_CP_*) as the minimum value of the set of ratios between each constituent’s percentage abundance in the mixture (*b_i_*) and its weight of evidence (WOE) NESIL (*c_i_*).

$$For\;\left\{b_{1},b_{2},\ldots ,b_{n}\right\}\;and\left\{c_{1},c_{2},\ldots ,c_{n}\right\}$$

(1) $${NESIL}_{CP}=min\left\{\frac{c_{1}}{b_{1}},\frac{c_{2}}{b_{2}},\ldots ,\frac{c_{n}}{b_{n}}\right\}$$

The model also yields a value for the normalized percentage of assessed constituents for dermal sensitization (*nPAC_ds_*). This value is calculated as the sum of the relative percentage abundance (*b_i_*) of constituents with sufficient data to classify dermal sensitization hazard and conclude a WOE NESIL (i.e., *c* ≠ *Ø*), divided by sum of the percentage abundance of all constituents in the mixture.

(2) $${nPAC}_{ds}=\frac{\sum_{i|c\neq \emptyset }b_{i}}{\sum b_{i}}$$

The current study used GC–MS percentage area under the curve (%AUC) values for all detected constituents as estimates of relative constituent abundance, summing to a total of 100% for each test material. As such, the *nPAC_ds_* values in this study are equivalent to a simple sum of the percentage of assessed constituents. However, in situations where the total sum may be greater or less than 100%, for example, if average abundance from multiple material lots were used for each constituent, the percentage of assessed constituents was normalized to the total sum of all constituents.

### 2.4. GARDskin DR

Testing was performed in accordance with the experimental setup of the GARDskin DR described in Gradin et al. (2021) [24], which is based on the validated GARDskin protocols outlined in OECD TG 442E [20]. The difference between the two protocols is that while GARDskin utilizes a single concentration to determine hazard, the GARDskin DR includes additional concentrations to construct a dose–response curve for prediction of continuous potency. In brief, the test system consists of a human myeloid dendritic-like cell line (SenzaCells™ ATCC Depository PTA-123875, Senzagen AB, Lund, Sweden) where each test chemical is exposed to the cells in an appropriate vehicle. Test chemicals are prepared for cellular stimulations according to established GARDskin protocols including solubility and cytotoxicity assessment, selection of vehicle and identification of top input concentration (GARD input concentration) for the dose–response curve (decrease of approximately 10% in cell viability compared to unstimulated controls). Following determination of the top input concentration, five additional concentrations were generated using a serial dilution factor of 0.5. Each test chemical was evaluated in two independent experiments, yielding biological replicates for each concentration.

After 24 h of exposure, cells were harvested and total RNA was extracted and subjected to quality control. Gene expression levels of the GARD prediction signature, comprising a predefined set of biomarker genes previously described in the literature [32], were quantified using NanoString nCounter technology (NanoString Technologies, Seattle, WA, USA). Raw gene expression data were processed using the GARD data analysis application as described in the supporting documents for the OECD TG for GARDskin [20]. Decision values (DVs) were generated using a support vector machine model and assigned to each test chemical and concentration.

To determine dose–response potency, the lowest concentration eliciting a positive classification response, termed the cDV_0_-value, was identified, as described by Gradin et al. (2021) [24]. Briefly, DVs were plotted against stimulation concentrations to evaluate concentration–response relationships. For substances showing a concentration-dependent increase in DVs that crossed the binary classification threshold within the tested range, the cDV_0_ was estimated by linear interpolation between the two concentrations bordering the threshold.

Following determination of chemical-specific cDV_0_ values, these were converted to NESIL values (µg/cm^2^) using a linear regression model as described in Gradin et al. (2024) [13]. The model was trained on a composite potency metric developed to address uncertainties in both human and animal reference data, using an error-in-variable approach integrating LLNA EC3 values with available human NESIL data.

### 2.5. LLNA Potency Measures

LLNA potency measures were collected from the published literature [33,34,35,36,37] (see Table A9 in Appendix B for details). LLNA EC3 values were converted to NESIL estimates in µg/cm^2^ by multiplying the percentage values by 250 [38].

### 2.6. NESIL Comparison and Statistics

Association between NESIL derivation methods was assessed using Spearman’s rank correlation coefficient. Agreement was evaluated using Bland–Altman analysis on log10-transformed NESIL values to estimate mean bias and 95% limits of agreement. Overall differences among methods were examined using the Friedman test, with concordance quantified by Kendall’s W. Post hoc pairwise comparisons were conducted following a significant Friedman test on log10-transformed NESIL values using two-sided Wilcoxon signed-rank tests with a Holm correction for multiple comparisons. Statistical analyses were conducted in Python (version 3.14.0). Figures showing NESIL comparisons and Bland–Altman plots were created using Python (version 3.14.0). An independent statistical review was conducted by an expert statistician who reviewed the dataset and confirmed the reproducibility and appropriateness of all statistical methods in R (version 4.5.2) using the blandr (v0.6.0) and DescTools (v0.99.60) packages. The final analysis and interpretation remain the responsibility of the authors.

## 3. Results

### 3.1. GC–MS Analysis

Constituent profiles for compounds appearing at ≥1% in the 8 assessed materials are shown in Table A1, Table A2, Table A3, Table A4, Table A5, Table A6, Table A7 and Table A8 in Appendix A (see Supplementary Materials Figures S1–S8 for chromatograms and full composition). The main constituents identified in cedarwood oil are alpha-cedrene (32%), cis-thujopsene (20%), cedrol (11%), and widdrol (10%). The main constituent(s) in cinnamon oil is trans-cinnamaldehyde (64%), in clove bud oil are eugenol (73%) and eugenol acetate (15%), in geranium oil are citronellol (32%), geraniol (11%), and citronellyl formate (10%), in lavender oil are linalool (32%) and linalyl acetate (28%), in lemongrass oil are geranial (41%) and neral (31%), in spearmint oil are carvone (59%) and limonene (22%), and in tea tree oil are terpinene-4-ol (41%), gamma-terpinene (21%), and alpha-terpinene (11%).

### 3.2. Component-Based Prediction Model

The predicted primary exposure-limiting constituent in each material determined by the CP is shown in Table 2 along with its weight of evidence NESIL and relative percentage in the raw material. The model predicts that dermal sensitization properties are driven by cedrene (alpha-cedrene and beta-cedrene isomers combined) in cedarwood oil, trans-cinnamaldehyde in cinnamon bark oil, eugenol in clove bud oil, citronellyl formate in geranium oil, linalyl acetate in lavender oil, citral (neral and geranial isomers combined) in lemongrass oil, and alpha-terpinene in tea tree oil.

The concentrations of the exposure-limiting constituents in the eight test materials range from 10% to 73% (median: 48.5%) and constituent Research Institute for Fragrance Materials (RIFM) WOE NESIL values range 590–10,000 µg/cm^2^ (median: 3050 µg/cm^2^). nPAC_ds_ values range 26–92% (median: 75%). NESIL_CP_ values range 900–64,000 µg/cm^2^ (median: 8700 µg/cm^2^).

### 3.3. GARDskin DR

Of the eight materials that were evaluated in the GARDskin DR assay, seven generated a dose–response curve, i.e., were identified as skin sensitizers, while one was identified as a non-sensitizer, i.e., the DV did not exceed the threshold at any tested concentration.

**Figure 1:**
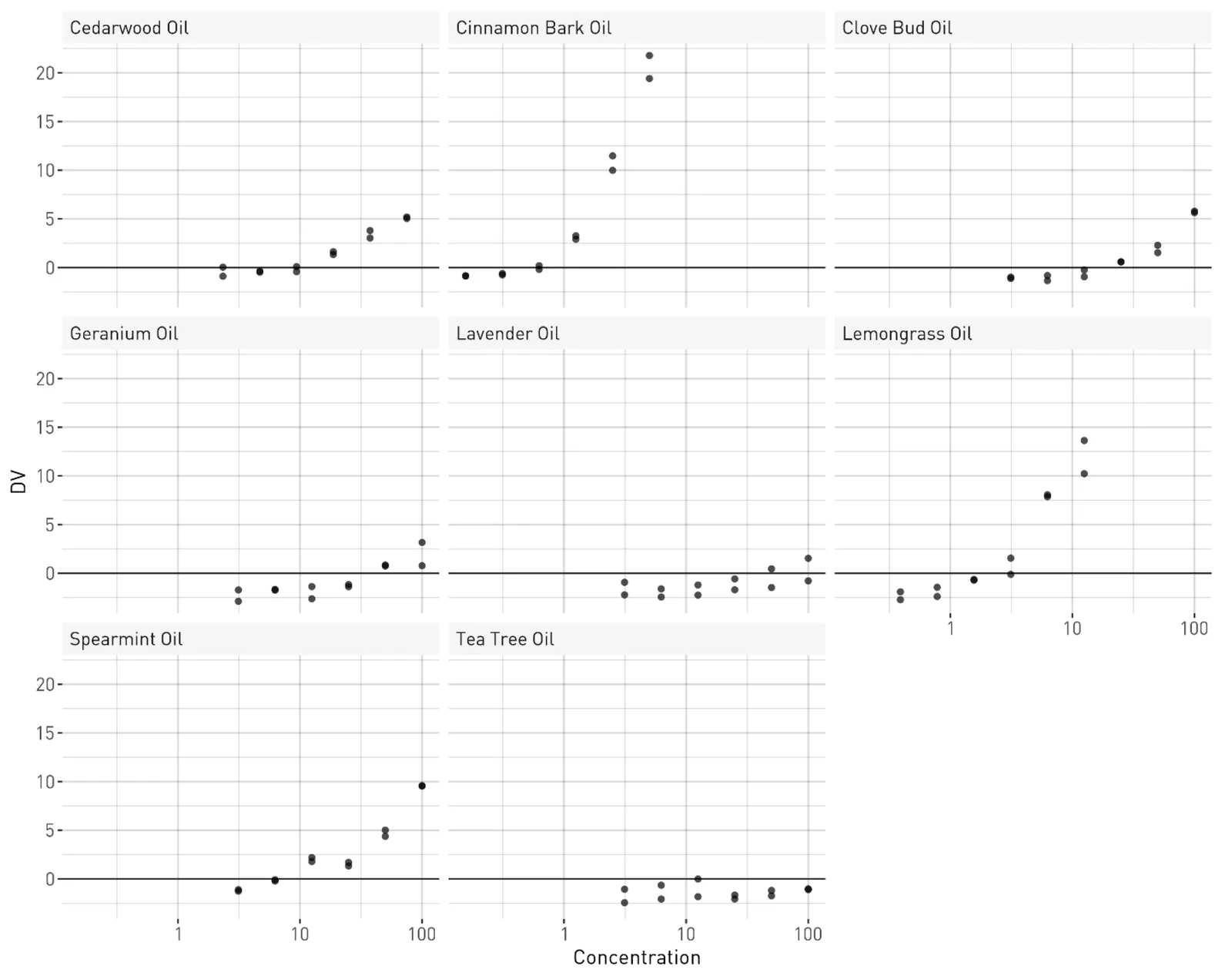
Dose–response relationships for the examined materials. Points represent measured DVs at respective concentrations (µg/mL). Each graph is marked with a bolded line representing the cDV_0_ binary sensitization classification threshold.

For substances with an identified cDV_0_, this value was entered into a linear regression model describing the relationship between experimentally derived cDV_0_ values and skin sensitizing potency, yielding continuous potency predictions in µg/cm^2^. In Table 3, each cDV_0_ with corresponding predicted NESIL value can be seen. Additionally, assayed concentrations, vehicle, and determined solubility are shown. Furthermore, Figure 1 illustrates all the experimentally derived dose–response curves with corresponding DV/concentration relationship for each material.

### 3.4. NESIL Comparisons

**Figure 2:**
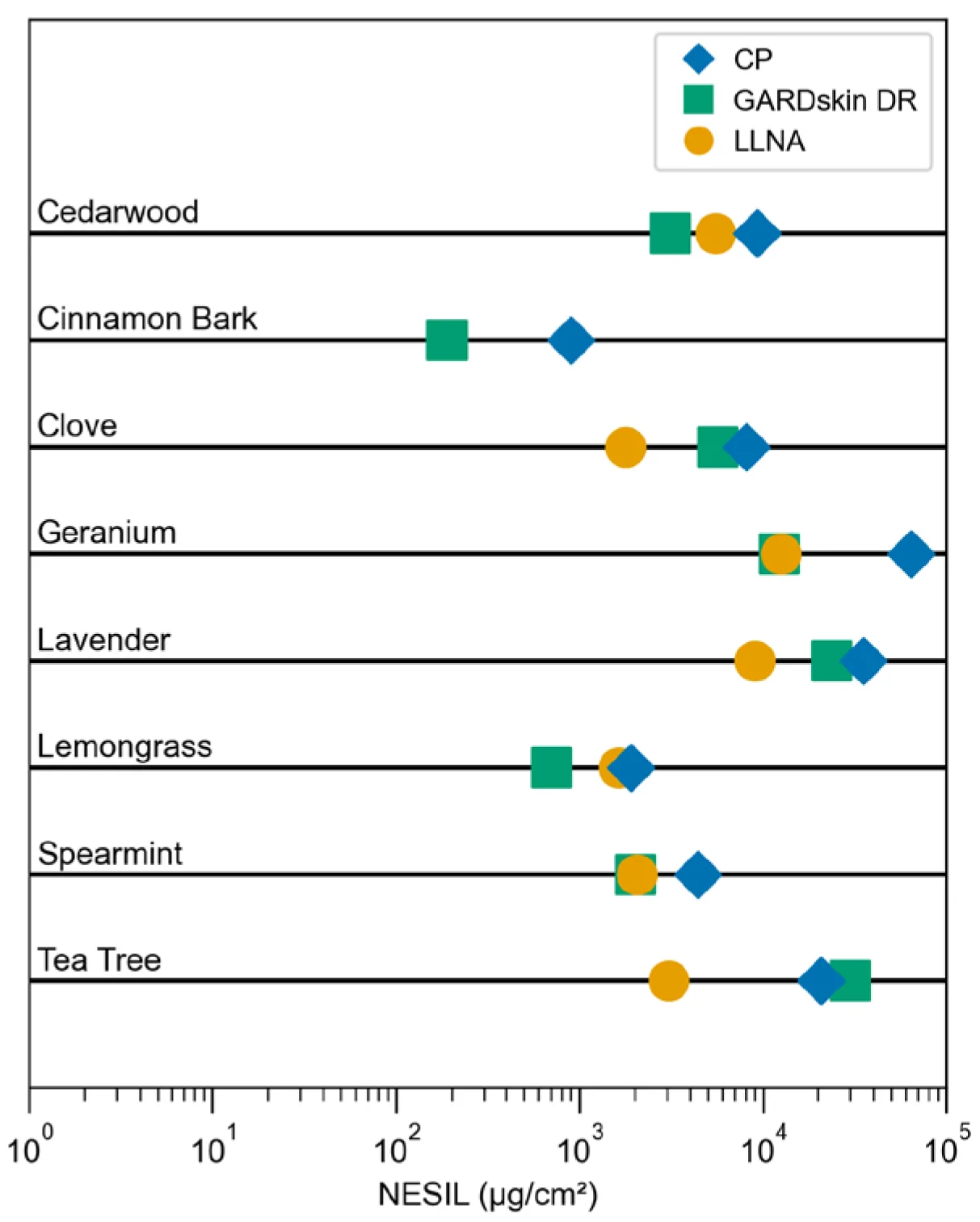
Graphical comparison of NESIL values from CP, GARDskin DR, and LLNA for the examined materials on a logarithmic scale.

**Figure 3:**
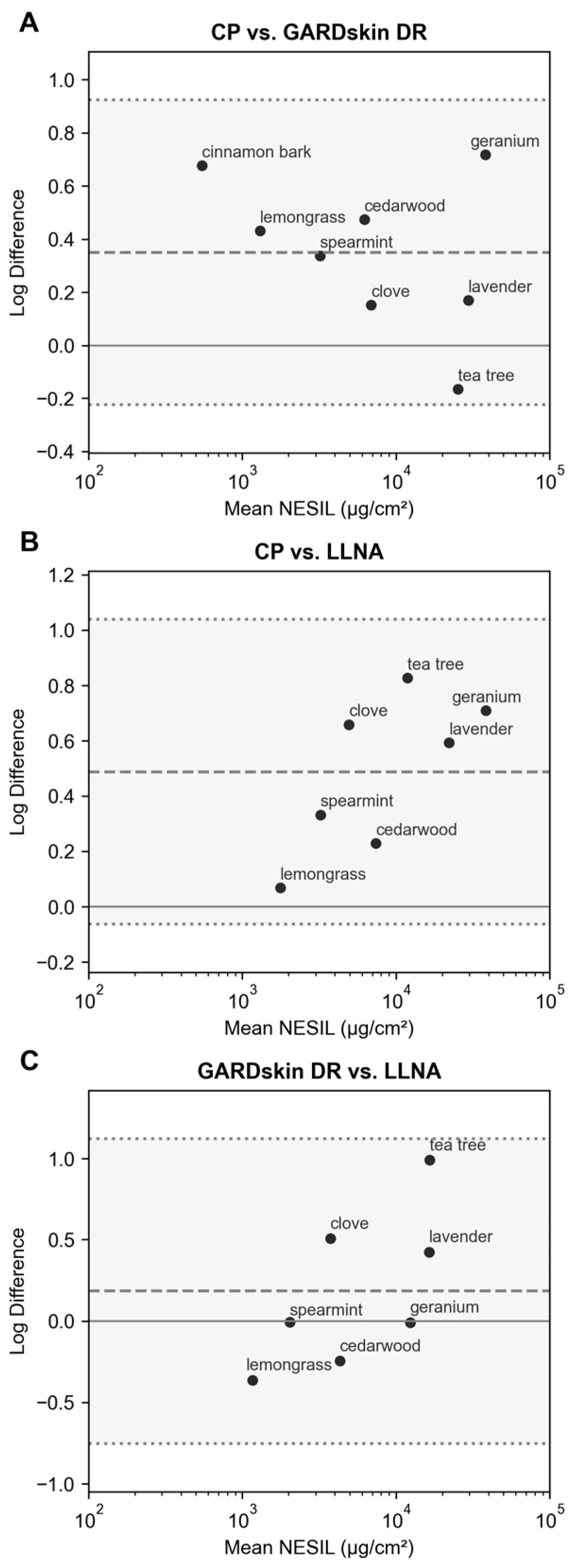
Bland-Altman plots for the examined materials showing pairwise comparisons of agreement between (**A**) CP vs. GARDskin DR, (**B**) CP vs. LLNA, and (**C**) GARDskin DR vs. LLNA. Mean bias is shown by the dashed lines, 95% limits of agreement are shown by the fine dotted lines, and zero log difference is shown by the solid lines.

Side-by-side comparisons of NESIL values derived from CP, GARDskin DR, and LLNA are shown in Figure 2 and Table 4. For the statistical analyses, the “n/a” cinnamon bark oil LLNA result was treated as missing, >12,500 µg/cm^2^ for geranium oil’s LLNA was treated as 12,500 µg/cm^2^, and >30,000 µg/cm^2^ for tea tree oil’s GARDskin DR was treated as 30,000 µg/cm^2^. Spearman correlations were calculated using pairwise complete data and show that NESIL_CP_ values correlate well with NESIL_GARD_ (rho = 0.881, *n* = 8, *p* = 0.007) and NESIL_LLNA_ (rho = 0.929, *n* = 7, *p* = 0.007), respectively. However, the correlation between NESIL_GARD_ and NESIL_LLNA_ is moderate and not statistically significant (rho = 0.607, *n* = 7, *p* = 0.17). Bland–Altman plots are shown in Figure 3. Log-transformed bias and 95% limits of agreement values are as follows: CP vs. GARDskin DR: 0.35 (−0.22–0.92), CP vs. LLNA: 0.49 (−0.06–1.04), and GARDskin DR vs. LLNA: 0.18 (−0.75–1.12). A sensitivity analysis excluding tea tree oil resulted in a reduction in log-transformed bias and narrowing limit-of-agreement values for GARDskin vs. LLNA: 0.05 (−0.64, 0.74), but made minor changes in the other pairwise comparisons (Table S2). Freidman’s test, post hoc pairwise Wilcoxon signed-rank tests with Holm adjustment, and Kendall’s W were evaluated for the seven materials with complete data only, excluding cinnamon bark oil. Friedman’s test showed a statistically significant difference among the methods (χ^2^(2) = 7.71, *p* = 0.021). Post hoc pairwise Wilcoxon signed-rank tests with Holm adjustment showed that NESIL_CP_ differed significantly from NESIL_LLNA_ (p_adjusted_ = 0.047), while other pairwise comparisons were not significant. Overall, strong statistical concordance was observed between the methods as indicated by Kendall’s W (W = 0.857, *p* = 0.017). Sensitivity analyses excluding geranium oil produced similar Spearman correlations, Bland–Altman estimates, and Kendall’s W (Table S2). While the overall difference between methods detected in Friedman’s test remained during the sensitivity analysis (χ^2^(2) = 6.33, *p* = 0.042), the Holm-adjusted CP-LLNA post hoc comparison was no longer significant (*p* = 0.094). This indicates that the correlation and concordance findings for this small sample set are robust, whereas the specific CP-LLNA difference was sensitive to geranium oil. Given the limited number of materials tested in the current study, findings from all statistical analyses should be interpreted descriptively.

## 4. Discussion

Overall, the findings from this study show concordance in the rank ordering between dermal sensitization potency values derived using the CP, GARDskin DR, and LLNA approaches. Results show strong correlation between the NESIL values from the CP compared to both GARDskin DR and LLNA. The NESIL_CP_ values found in this study are numerically greater than their corresponding NESIL_GARD_ and NESIL_LLNA_, but this observation was sensitive to geranium oil, and evidence presented in this study does not establish a robust systematic difference between the CP compared to GARDskin DR or LLNA. Additionally, results of this study show a non-significant rank correlation between GARDskin DR and LLNA, despite having overall numerical similarity. In fact, the numerical difference between GARDskin DR and LLNA was largely driven by tea tree oil. This indicates the importance of weighing the findings from the numerical agreement analyses with the statistical significance of findings when interpreting the results of this study.

Due to the small sample (*n* = 7 or 8), all of the statistical findings from this study should be interpreted with caution as they are potentially unstable and insufficient to support generalizable findings. Rather, these analyses are intended to be interpreted descriptively as objective measures of the agreement between the CP, GARDskin DR, and LLNA outputs reported in this exploratory proof-of-concept study. Indeed, an analysis of a larger sample set demonstrated statistically significant Spearman’s rank correlation between GARDskin DR and LLNA for discrete compounds [13]. Likewise, the strong rank correlation in pairwise tests with CP as well as the concordance findings from the omnibus tests may become non-significant with the addition of other essential oil samples. With that said, the NESIL results reported in this study are informative and provide preliminary insights for research surrounding the utility of GARDskin DR and CP for determining the dermal sensitization potency of essential oils.

Previous research has determined that the standard deviation of the log-transformed LLNA EC3 is 0.15–0.31 [47,48]. In an evaluation of reproducibility, the residual standard deviation of the GARDskin DR assay was reported to be 1.8-fold [12]. While the NESIL_CP_ values in this study are often greater than their corresponding NESIL_GARD_ and NESIL_LLNA_ values, the absolute log difference between these values is <0.2 for 4/8 of the materials tested. This is the case for NESIL_CP_ vs. NESIL_GARD_ in clove bud oil, lavender oil, and tea tree oil and for NESIL_CP_ vs. NESIL_LLNA_ in lemongrass oil. Additionally, the absolute log difference is <0.2 for the NESIL_GARD_ vs. NESIL_LLNA_ in spearmint oil and geranium oil. For these cases, close agreement between dermal sensitization potency values in only two of the test methods provides an interesting opportunity explore the potential reasons for variance in the third method and to gauge the practical significance of its difference.

The NESIL_GARD_ and NESIL_LLNA_ values for spearmint oil are nearly equivalent (2020 and 2050 µg/cm^2^, respectively), whereas the NESIL_CP_ is over twofold higher (4400 µg/cm^2^). The CP determined that carvone is the exposure-limiting constituent and is found at a concentration of 60% in the sample of spearmint oil tested. Spearmint oil specifically contains L-carvone (CAS 6485-40-1), for which RIFM has reported a WOE NESIL of 2600 µg/cm^2^ based on human data [45]. Results from three separate LLNA studies show NESIL_LLNA_ values for carvone of 2675–3250 µg/cm^2^ [45,49,50]. With regard to L-carvone in GARDskin DR, Gradin et al. report a NESIL_GARD_ of 1560 µg/cm^2^ [13] and Lee et al. report 2680 µg/cm^2^ [23]. From these data, we see that the dermal sensitizing potency of spearmint oil in GARDskin DR and LLNA is as sensitizing, if not slightly more, than its constituent parts alone. This is intriguing because the dermal sensitization effect of a mixture is conventionally believed to be driven by a single constituent or constituent group [5]. While the tested sample of spearmint oil does contain other constituents with sensitizing potential (limonene, 1,8-cineole, myrcene, and beta-pinene), concentrations of these constituents are below levels that would be expected to be sensitizing. Further investigation will be necessary to determine if the dermal sensitization potentiation observed in LLNA and GARDskin DR testing of spearmint oil compared to L-carvone is significant, and if so, by what mechanism it occurs.

The NESIL_CP_ and NESIL_LLNA_ values for lemongrass oil are similar (1900 and 1625 µg/cm^2^, respectively), whereas the NESIL_GARD_ is somewhat lower (703 µg/cm^2^). The CP determined that citral is the exposure-limiting constituent and is found at a concentration of 73% in the tested sample of lemongrass oil. RIFM has reported a WOE NESIL of 1400 µg/cm^2^ for citral based on LLNA and human data [44], whereas GARDskin DR shows a NESIL of about 330 µg/cm^2^ for citral [13,23]. The relative dermal sensitization potency of citral in GARDskin DR and LLNA closely mirrors the pattern we see for lemongrass oil in the current study. Again, this is intriguing because despite the fact that the tested lemongrass oil contains dermal sensitizing compounds other than citral (e.g., geraniol, geranyl and neryl acetate, isocitral, linalool, and 6-methyl-5-hepten-2-one), these compounds do not appear to enhance the dermal sensitization potency of the overall mixture.

The NESIL_CP_ and NESIL_GARD_ values for clove bud oil are relatively similar (8100 and 5690 µg/cm^2^, respectively), whereas the NESIL_LLNA_ is over threefold smaller (1775 µg/cm^2^). The CP determined that eugenol is the exposure-limiting constituent and is found at a concentration of 73% in the tested sample of clove bud oil. Indeed, eugenol is the only confirmed dermal sensitizer in clove bud oil. The RIFM WOE NESIL reported for eugenol is 5900 µg/cm^2^ based on human data [41]. The same report cites a weighted mean NESIL_LLNA_ of 2827 µg/cm^2^ [41]. NESIL_LLNA_ values for eugenol reported in the literature ar 1350–6275 µg/cm^2^ [35,51]. Considering the range of potency values reported in the literature for eugenol and the fact that only one LLNA study could be identified for clove bud oil, it is reasonable to assume that repeated LLNA testing of clove bud oil may yield a range of NESIL values similar to what we see with eugenol. If this is the case, then it would be expected that the NESIL_LLNA_ range for clove bud oil would extend into the NESIL_CP_ and NESIL_GARD_ values determined in the current study. Together with the roughly proportional correlation we see between the published NESIL_GARD_ value for eugenol (2824 µg/cm^2^) [13,24] and the clove bud oil findings from this study when adjusting for the concentration of eugenol in clove bud oil, we are confident that the NESIL_GARD_ of 5690 µg/cm^2^ reported here is a sufficiently conservative POD for the dermal sensitization of clove bud oil.

For lavender oil, the NESIL_CP_ and NESIL_GARD_ values are relatively similar (35,300 and 23,800 µg/cm^2^, respectively) whereas the NESIL_LLNA_ is two- to threefold smaller (9000 µg/cm^2^). Linalyl acetate is determined by the CP to be the exposure-limiting constituent and is found at a concentration of 28% in the tested sample of lavender oil. The RIFM WOE NESIL for linalyl acetate is 10,000 µg/cm^2^ based on human data, with NESIL_LLNA_ values of 400–6250 µg/cm^2^ [43,52]. A NESIL_GARD_ value for linalyl acetate could not be found in the published literature. Again, based on the range of NESIL_LLNA_ values for linalyl acetate reported in the literature, it may reasonably be expected for repeated LLNA testing of lavender oil to yield a range of values that extends into the to the NESIL_CP_ and NESIL_GARD_ values determined in this study.

The NESIL_CP_ and NESIL_GARD_ values for tea tree oil are relatively similar (20,600 and >30,000 µg/cm^2^, respectively) and far exceed the NESIL_LLNA_ (3066 µg/cm^2^). Tea tree oil is classified as a CLP 1B skin sensitizer based on results from a Draize HRIPT showing a 1% (3/309 subjects) sensitization-induction response rate with occlusive applications of 5%, 25%, and 100% tea tree oil and four LLNA studies showing EC3 values of 4.4–25.5% (NESIL: 1100–6375 µg/cm^2^) [37,53,54]. In a 2025 opinion from the Scientific Committee on Consumer Safety (SCCS) on tea tree oil, the lowest EC3 value was selected from these LLNA studies as a POD (4.4%, NESIL = 1100 µg/cm^2^) despite the test material in this particular study being oxidized tea tree oil and it being known that oxidized tea tree oil is more sensitizing than fresh tea tree oil [53,55]. The current study used a NESIL_LLNA_ value converted from the geometric mean of the EC3 values from the four available LLNA studies on tea tree oil (3066 µg/cm^2^), but still, this value is nearly an order-of-magnitude lower than the NESIL_GARD_ (>30,000 µg/cm^2^) and NESIL_CP_ (20,600 µg/cm^2^). Alpha-terpinene is the exposure-limiting constituent determined by the CP and is present at 11% in the sample of tea tree oil tested in the current study. The single LLNA study on alpha-terpinene yielded a NESIL_LLNA_ of 2225 µg/cm^2^ [50,56], which is in alignment with the RIFM WOE NESIL of 2200 µg/cm^2^ for alpha-terpinene [46]. At this point in time, alpha-terpinene has not been evaluated in GARDskin DR.

Relatively few of the constituents in the tested sample of tea tree oil have sufficient data to determine dermal sensitization potency (nPAC_ds_ = 26%), and perhaps a constituent other than alpha-terpinene may be driving the dermal sensitizing effect of tea tree oil. However, it is worth noting that tea tree oil has been found to be non-sensitizing in the in vitro KeratinoSens assay and several in vivo assays (guinea pig maximization test, human maximization test, and semi-occluded HRIPT) [53]. In the absence of a positive LLNA and Draize HRIPT, tea tree oil may have been classified as a dermal sensitizer based on the OECD TG 497 “two out of three” defined approach on skin sensitization, given the non-sensitizing KeratinoSens and GARDskin DR assays [21]. Further investigations exploring the dermal sensitization of tea tree oil using other in vitro methods and evaluating the potency of alpha-terpinene, gamma-terpinene, and terpinene-4-ol in GARDskin DR may help to reconcile the differences seen in the dermal sensitization potency of tea tree oil in the LLNA vs. the GARDskin DR and CP methods.

For cedarwood oil, NESIL_GARD_ < NESIL_LLNA_ < NESIL_CP_ (3120, 5500, and 9300 µg/cm^2^, respectively). Cedrene (alpha and beta isomers) are determined by the CP to be the exposure-limiting constituents in the tested sample of cedarwood oil and are found at a combined concentration of 38%. However, the tested sample of cedarwood oil also contains the dermal sensitizer cedrol at 11%, a level that is expected to have a significant sensitizing effect. Cedrene has a RIFM WOE NESIL of 3500 µg/cm^2^ based on human data but has not been tested in the LLNA [39]. Cedrol has a RIFM WOE NESIL of 2000 µg/cm^2^ based on human data and has been tested in a single LLNA showing a NESIL_LLNA_ of 4750 µg/cm^2^ [57]. Additionally, neither cedrol nor cedrene presently has GARDskin DR reference data, and we are unable to determine if these constituents exhibit a combinatorial effect on the dermal sensitization potency of cedarwood oil at this time. Furthermore, cedrene and cedrol are the only two constituents in the tested sample of cedarwood oil with sufficient data to determine a dermal sensitization potency value (nPAC_ds_ = 50%), and it is potentially possible that another constituent may be the primary driver in dermal sensitization potency. Conducting further testing to evaluate cedrene, cedrol, and cedarwood oil constituents in the GARDskin DR assay may provide additional insights.

For geranium oil, the NESIL_CP_ (64,000 µg/cm^2^) is greater than the NESIL_GARD_ and NESIL_LLNA_ (12,240 and >12,500 µg/cm^2^, respectively). It should be noted that this NESIL_LLNA_ value is based on a single LLNA study where the EC3 for geranium oil was found to be >50%, the highest concentration tested [35]. Citronellyl formate is determined by the CP to be the exposure-limiting constituent and is present at 10% in the tested sample of geranium oil. RIFM concluded a WOE NESIL of 6400 µg/cm^2^ for citronellyl formate based on human and LLNA studies conducted on citronellyl formate and its read-across material citronellyl butyrate [42]. Citronellyl formate and citronellyl butyrate have each been tested in a single LLNA showing NESIL_LLNA_ values of 8087 µg/cm^2^ and 6600 µg/cm^2^, respectively [42]. At this time, citronellyl formate has not been evaluated using GARDskin DR.

While we cannot completely exclude the possibility that another constituent in the tested sample of geranium oil may be driving the dermal sensitization potency, this seems unlikely considering that the dermal sensitization properties of most of the material constituents are well-described (nPAC_ds_ = 77%). Neither can we exclude the possibility that the presence of other dermal sensitizers in the tested sample of geranium oil (citronellol, geraniol, menthone, isomenthone, linalool, geranyl formate, geranyl butyrate, rose oxide, and citral) may potentiate its dermal sensitization effect in the GARDskin DR assay. It will be interesting to see in future testing if the potency of citronellyl formate in GARDskin DR compared to geranium oil is proportionate to the 10% concentration that this constituent is present in the test material.

The 64,000 µg/cm^2^ NESIL_CP_ for the tested sample of geranium oil is very high, especially considering that the maximum NESIL thresholds for LLNA and GARDskin DR are 25,000 and 30,000 µg/cm^2^, respectively. Indeed, the 64,000 µg/cm^2^ NESIL_CP_ for geranium oil reported in this study is well beyond the testable scale, and an upper NESIL_CP_ threshold needs to be defined for the CP model. It should be noted that NESIL values around this magnitude are not completely unheard of, for example, the WOE NESIL reported for both benzyl benzoate and cis-3-hexenyl benzoate is 59,000 µg/cm^2^ [58,59]. Additionally, the maximum exposure to a neat material in a CNIH study is approximately 61,000 µg/cm^2^ (0.3 g test material applied in a 25 mm Hill Top Chamber patch) [7]. With this said, the upper NESIL thresholds for preclinical and clinical testing are clearly skewed, especially when interpreted on a linear scale. The current study attempts to overcome this limitation by quantifying comparisons between NESIL values on a logarithmic scale. However, until a well-defined methodology to normalize sensitization potency outcomes between the LLNA, GARDskin DR, and CNIH is established, it must be understood that in the upper range, approximately >10,000 µg/cm^2^, NESIL values have wider variation between methods. This variation may impact statistical analyses of inter-method comparisons for dermal sensitizers in the “weak sensitizing” range, as indicated by a significant in difference in the Holm-adjusted CP and LLNA post hoc comparison that was no longer significant when geranium oil was removed.

It should also be noted that geranium oil is highly variable in composition. While the sample of geranium oil tested in this study aligns with an Egyptian chemotype [60], it is unknown if differences in the African chemotype tested in the reference LLNA [35] significantly impact the dermal sensitization potency. Indeed, this is an important caveat for all of the essential oil LLNA reference values cited in the current analysis as NCSs are known to vary significantly based on their source [61], and their characterization is often poorly described in pharmacological and toxicological literature [62,63]. Significant compositional differences between test materials evaluated in the LLNA compared to the CP and GARDskin DR could impact the LLNA pairwise statistical comparisons and the omnibus tests. Additionally, the findings from the current study cannot be assumed to apply to essential oils from the same plant species, but different plant part, growing conditions, extraction method, country of origin, or any number of factors that may cause a significant difference in essential oil chemistry. While the chemistry of each essential oil tested in the current analysis is relatively reproducible between batches (unpublished data), to the authors’ knowledge a standardized criterion of sufficient similarity for dermal sensitization read across has yet to be established for mixtures. Such a criterion would be important for determining if the NESIL values reported in the current study are sample-specific or can be generalized, for example, to lavender oil or tea tree oil as a category.

For cinnamon bark oil, the NESIL_CP_ (900 µg/cm^2^) is greater than the NESIL_GARD_ (189 µg/cm^2^) by a factor of about 4.8-fold (Δlog = +0.68). No LLNA was available for cinnamon bark oil. Trans-cinnamaldehyde is the exposure-limiting constituent determined by the CP and is present in the tested sample of cinnamon bark oil at 64%. The WOE NESIL for trans-cinnamaldehyde is 590 µg/cm^2^ based on human data, and the weighted mean NESIL_LLNA_ is 262 µg/cm^2^, as reported by RIFM in their safety assessment for trans-cinnamaldehyde [40]. The same assessment also reports a dermal sensitization LOAEL of 775 µg/cm^2^ for trans-cinnamaldehyde due to the induction of sensitization in 5/41 volunteers in a confirmatory HRIPT. In GARDskin DR trans-cinnamaldehyde showed a NESIL of 159 µg/cm^2^ [13,24]. Based on the relative congruence between GARDskin DR and LLNA for trans-cinnamaldehyde, it is likely that NESIL values for cinnamon bark oil from these test methods may be similar. As such, this example from the current study suggests that the conservative nature of GARDskin DR for trans-cinnamaldehyde may be preserved in the testing of cinnamon bark oil. Despite this, a more permissive WOE NESIL for trans-cinnamaldehyde has been concluded based on high-quality testing for dermal sensitization in humans as well as the extensive and well-documented history of safe use for this compound when diluted appropriately. It is not entirely impossible that a constituent of unknown dermal sensitization potency in cinnamon bark oil may be driving the dermal sensitization effect in GARDskin DR. However, this seems unlikely since the dermal sensitization properties of most of the constituents in the tested sample of cinnamon bark oil (nPAC_ds_ = 82%) have been well characterized. Altogether, this indicates that the CP may be a more relevant approach than GARDskin DR for deriving a NESIL for the tested sample of cinnamon bark oil.

## 5. Conclusions

In conclusion, an overall concordance in material ranking was observed in this comparative analysis between the outputs of three separate methods to measure dermal sensitization potency for the eight essential oils evaluated. With that said, the pairwise correlations between methods in this small sample set were heterogenous, with strong significant correlations being observed between the ranked outputs from the CP compared to GARDskin DR and LLNA. The moderate correlation observed between GARDskin DR and LLNA was not statistically significant. Given the small sample, no statistical findings from this study are generalizable and should be interpreted descriptively. However, while descriptive and limited in scope, these findings provide preliminary evidence related to the applicability of GARDskin DR and CP for estimating the dermal sensitization potency of the selected essential oils. Further GARDskin DR testing of other mixtures with well-characterized dermal sensitization effects is needed to confirm these findings. Thorough characterization of the chemical composition of each mixture, as well as the sensitizing effects of each mixture’s constituents, will be needed to evaluate the conditions under which the dermal sensitization potency of a mixture may be different from its separate components. Additionally, it may be valuable to examine the applicability of essential oils in SARA-ICE to explore the potential of obtaining an additional comparison of dermal sensitization potency. As the use of NAMs becomes increasingly accepted for defining the dermal sensitization of materials, the findings from this and future investigations may have important implications for how risk assessors weigh dermal sensitization data for mixtures and their discrete parts.

## Figures and Tables

**Table 1:** Identity, source, origin, and extraction method of the test materials.

Material Name	CAS	Botanical Name	Plant Part	Country of Origin	Extraction Method
Cedarwood Oil	8000-27-9	*Juniperus virginiana*	Wood	United States	Steam Distillation
Cinnamon Bark Oil	8007-80-5	*Cinnamomum zeylanicum*	Bark	Sri Lanka	Steam Distillation
Clove Bud Oil	8000-34-8	*Eugenia caryophyllata*	Bud	Indonesia	Steam Distillation
Geranium Oil	8000-46-2	*Pelargonium graveolens*	Aerial Parts	Egypt	Steam Distillation
Lavender Oil	90063-37-9	*Lavandula angustifolia*	Aerial Parts	Bulgaria	Steam Distillation
Lemongrass Oil	8007-02-1	*Cymbopogon citratus*	Aerial Parts	India	Steam Distillation
Spearmint Oil	8008-79-5	*Mentha spicata*	Leaf, Stem	India	Steam Distillation
Tea Tree Oil	68647-73-4	*Melaleuca alternifolia*	Aerial Parts	Kenya	Steam Distillation

**Table 2:** Relative abundance and WOE NESIL values for dermal sensitization exposure-limiting constituents in the 8 test materials along with material nPAC_ds_ and NESIL_CP_ values.

Material Name	Exposure-Limiting Constituent *	Constituent Concentration (%)	Constituent WOE NESIL (µg/cm^2^)	nPAC_ds_	NESIL_CP_ (µg/cm^2^)
Cedarwood Oil	cedrene	37.6%	3500 [39]	50%	9300
Cinnamon Bark Oil	trans-cinnamaldehyde	64.2%	590 [40]	82%	900
Clove Bud Oil	eugenol	73.0%	5900 [41]	73%	8100
Geranium Oil	citronellyl formate	10.0%	6400 [42]	77%	64,000
Lavender Oil	linalyl acetate	28.3%	10,000 [43]	73%	35,300
Lemongrass Oil	citral	72.6%	1400 [44]	92%	1900
Spearmint Oil	carvone	59.4%	2600 [45]	90%	4400
Tea Tree Oil	alpha-terpinene	10.7%	2200 [46]	26%	20,600

* The exposure-limiting constituent is the component predicted by the CP model to drive the dermal sensitization potency of the material. Other dermal sensitizers present in the material do not contribute to the CP model’s dermal sensitization potency prediction.

**Table 3:** Summary of GARDskin DR results and study details for the evaluated materials.

Material Name	cDV_0_ (µg/mL)	NESIL_GARD_ (95% CI) (µg/cm^2^)	Stimulation Concentration (µg/mL)	Vehicle	Determined In-Well Solubility (µg/mL)
Cedarwood Oil	10.3	3120 (2125, 4580)	2.3–75	DMSO	100
Cinnamon Bark Oil	0.622	189 (129, 277)	0.2–5.0	DMSO	100
Clove Bud Oil	18.8	5690 (3880, 8360)	3.1–100	DMSO	100
Geranium Oil	40.3	12,240 (8337, 17,971)	3.1–100	DMSO	100
Lavender Oil	78.4	23,800 (16,200, 34,900)	3.1–100	DMSO	100
Lemongrass Oil	2.32	703 (479, 1032)	0.4–12.5	DMSO	100
Spearmint Oil	6.66	2020 (1376, 2966)	3.1–100	DMSO	100
Tea Tree Oil	NS	NS	3.1–100	DMSO	100

NS, non-sensitizing.

**Table 4:** NESIL values from the CP, GARDskin DR, and LLNA for the examined materials.

Material Name	NESIL_CP_ (µg/cm^2^)	NESIL_GARD_ (µg/cm^2^)	NESIL_LLNA_ (µg/cm^2^)
Cedarwood Oil	9300	3120	5500
Cinnamon Bark Oil	900	189	n/a
Clove Bud Oil	8100	5690	1775
Geranium Oil	64,000	12,240	>12,500
Lavender Oil	35,300	23,800	9000
Lemongrass Oil	1900	703	1625
Spearmint Oil	4400	2020	2050
Tea Tree Oil	20,600	>30,000	3066

## Data Availability

The original contributions presented in this study are included in the article/Supplementary Materials. Further inquiries can be directed to the corresponding author.

## Supplementary Materials

The following supporting information can be downloaded at https://www.mdpi.com/article/10.3390/toxics14090800/s1. Figure S1: Cedarwood oil GC–MS chromatogram and chemical composition; Figure S2: Cinnamon bark oil GC–MS chromatogram and chemical composition; Figure S3: Clove bud Oil GC–MS chromatogram and chemical composition; Figure S4: Geranium oil GC–MS chromatogram and chemical composition; Figure S5: Lavender oil GC–MS chromatogram and chemical composition; Figure S6: Lemongrass oil GC–MS chromatogram and chemical composition; Figure S7: Spearmint oil GC–MS chromatogram and chemical composition; Figure S8: Tea tree oil GC–MS chromatogram and chemical composition; Table S1: Manufacture, GC–MS, and GARDskin DR test dates for the examined materials; Table S2: Results of the primary and sensitivity statistical comparisons between the CP, GARDskin DR, and LLNA outputs for the test materials.

## Abbreviations

The following abbreviations are used in this manuscript:

%AUC	percentage area under the curve
cDV_0_	minimal concentration required to exceed the binary classification in GARDskin (DV ≥ 0)
CNIH	confirmation of no induction in human
CP	component-based prediction model
DV	decision value
GARDskin DR	dose–response adaptation of the genomic allergen rapid detection for skin sensitizers
GC–MS	gas chromatography–mass spectrometry
GHS	Global Harmonized System of Classification and Labeling of Chemicals
HRIPT	human repeat insult patch test
LLNA	local lymph node assay
LogP	logarithm of octanol–water partition coefficient
MW	molecular weight
NAM	new approach methodology
NCS	natural complex substance
NESIL	no expected sensitization induction level
nPAC_ds_	normalized percentage of assessed constituents for dermal sensitization
OECD TG	Organisation for Economic Co-operation and Development test guideline
POD	point of departure
RI	retention index
RIFM	Research Institute for Fragrance Materials
SARA-ICE	skin allergy risk assessment—integrated chemical environment
WOE	weight of evidence

## Appendix A

Constituent profiles from GC–MS analysis of the eight test materials are shown below in Table A1, Table A2, Table A3, Table A4, Table A5, Table A6, Table A7 and Table A8, showing retention index (RI), common name, IUPAC identifier, CAS, and %AUC for compounds appearing at concentrations ≥ 1%. Molecular weight (MW) and XLogP3-AA (LogP) values were obtained from PubChem [64].

**Table A1:** Constituent profile from GC–MS analysis of cedarwood oil.

RI	Compound	IUPAC	CAS	%AUC	MW (g/mol)	LogP
1419	alpha-cedrene	(1S,2R,5S,7S)-2,6,6,8-tetramethyltricyclo[5.3.1.01,5]undec-8-ene	469-61-4	31.8%	204.35	4.6
1437	cis-thujopsene	(1aS,4aS,8aS)-2,4a,8,8-tetramethyl-1,1a,4,5,6,7-hexahydrocyclopropa[j]naphthalene	470-40-6	19.6%	204.35	4.8
1611	cedrol	(1S,2R,5S,7R,8R)-2,6,6,8-tetramethyltricyclo[5.3.1.01,5]undecan-8-ol	77-53-2	10.9%	222.37	3.9
1609	widdrol	(7S,9aS)-4,4,7,9a-tetramethyl-1,2,3,6,8,9-hexahydrobenzo[7]annulen-7-ol	6892-80-4	10.2%	222.37	4.1
1625	beta-cedrene	(1S,2R,5S,7S)-2,6,6-trimethyl-8-methylidenetricyclo[5.3.1.01,5]undecane	546-28-1	5.8%	204.35	4.9
1502	alpha-cuprenene	1-methyl-4-(1,2,2-trimethylcyclopentyl)cyclohexa-1,3-diene	29621-78-1	2.7%	204.35	4.8
1533	gamma-cuprenene	1-methyl-4-[(1S)-1,2,2-trimethylcyclopentyl]-1,4-cyclohexadiene	4895-23-2	1.8%	204.35	4.8
1480	gamma-gurjunene	(1R,3aR,4R,7R)-1,4-dimethyl-7-prop-1-en-2-yl-1,2,3,3a,4,5,6,7-octahydroazulene	22567-17-5	1.6%	204.35	5.3
1503	pseudowiddrene	(4aS)-4,4,4a,7-tetramethyl-3,5,8,9-tetrahydro-2H-benzo[7]annulene	32540-28-6	1.3%	204.35	4.3
1507	cuparene	1-methyl-4-[(1R)-1,2,2-trimethylcyclopentyl]benzene	16982-00-6	1.3%	202.33	5.5
1505	alpha-chamigrene	(6R)-1,5,5,9-tetramethylspiro[5.5]undeca-1,9-diene	19912-83-5	1.1%	204.35	4.4

**Table A2:** Constituent profile from GC–MS analysis of cinnamon bark oil.

RI	Compound	IUPAC	CAS	%AUC	MW (g/mol)	LogP
1277	trans- cinnamaldehyde	(E)-3-phenylprop-2-enal	104-55-2	64.2%	132.16	1.9
1415	trans-beta- caryophyllene	(1R,4E,9S)-4,11,11-trimethyl-8-methylidenebicyclo[7.2.0]undec-4-ene	87-44-5	5.8%	204.35	4.4
1355	eugenol	2-methoxy-4-(prop-2-en-1-yl)phenol	97-53-0	5.4%	164.20	2.0
1027	beta-phellandrene	3-methylidene-6-propan-2-ylcyclohexene	555-10-2	3.1%	136.23	3.4
1099	linalool	3,7-dimethylocta-1,6-dien-3-ol	78-70-6	2.9%	154.25	2.7
1021	para-cymene	1-methyl-4-propan-2-ylbenzene	99-87-6	2.4%	134.22	4.1
1003	alpha-phellandrene	2-methyl-5-propan-2-ylcyclohexa-1,3-diene	99-83-2	2.1%	136.23	3.2
929	alpha-pinene	2,6,6-trimethylbicyclo[3.1.1]hept-2-ene	80-56-8	2.0%	136.23	2.8
1440	trans-cinnamyl acetate	[(E)-3-phenylprop-2-enyl] acetate	103-54-8	1.5%	176.21	2.3
1450	alpha-humulene	(1E,4E,8E)-2,6,6,9-tetramethylcycloundeca-1,4,8-triene	6753-98-6	1.0%	204.35	4.5
1026	limonene	(4R)-1-methyl-4-prop-1-en-2-ylcyclohexene	5989-27-5	1.0%	136.23	3.4

**Table A3:** Constituent profiles from GC–MS analysis of clove bud oil.

RI	Compound	IUPAC	CAS	%AUC	MW (g/mol)	LogP
1355	eugenol	2-methoxy-4-(prop-2-en-1-yl)phenol	97-53-0	73.0%	164.20	2.0
1511	eugenol acetate	(2-methoxy-4-prop-2-enylphenyl) acetate	93-28-7	15.1%	206.24	2.3
1415	trans-beta- caryophyllene	(1R,4E,9S)-4,11,11-trimethyl-8-methylidenebicyclo[7.2.0]undec-4-ene	87-44-5	8.7%	204.35	4.4
1450	alpha-humulene	(1E,4E,8E)-2,6,6,9-tetramethylcycloundeca-1,4,8-triene	6753-98-6	1.4%	204.35	4.5
1576	caryophyllene oxide	(1R,4R,6R,10S)-4,12,12-trimethyl-9-methylidene-5-oxatricyclo[8.2.0.04,6]dodecane	1139-30-6	0.4%	220.35	3.6

**Table A4:** Constituent profile from GC–MS analysis of geranium oil.

RI	Compound	IUPAC	CAS	%AUC	MW (g/mol)	LogP
1227	citronellol	3,7-dimethyloct-6-en-1-ol	106-22-9	32.1%	156.26	3.2
1250	geraniol	(2E)-3,7-dimethylocta-2,6-dien-1-ol	106-24-1	11.8%	154.25	2.9
1270	citronellyl formate	3,7-dimethyloct-6-en-1-yl formate	105-85-1	10.0%	184.27	3.8
1161	isomenthone	cis-(2S,5S)-5-methyl-2-propan-2-ylcyclohexan-1-one	491-07-6	6.3%	154.25	2.7
1623	10-epi-gamma- eudesmol	2-[(2R,4aS)-4a,8-dimethyl-2,3,4,5,6,7-hexahydro-1H-naphthalen-2-yl]propan-2-ol	15051-81-7	5.0%	222.37	3.4
1096	linalool	3,7-dimethylocta-1,6-dien-3-ol	78-70-6	4.4%	154.25	2.7
1294	geranyl formate	(2E)-3,7-Dimethylocta-2,6-dien-1-yl formate	105-86-2	3.6%	182.26	3.5
1476	germacrene d	(1E,6E,8S)-1-methyl-5-methylidene-8-propan-2-ylcyclodeca-1,6-diene	23986-74-5	1.6%	204.35	4.7
1550	geranyl butyrate	(2E)-3,7-Dimethylocta-2,6-dien-1-yl butanoate	106-29-6	1.5%	224.34	4.3
1690	geranyl tiglate	[(2E)-3,7-dimethylocta-2,6-dienyl] (E)-2-methylbut-2-enoate	7785-33-3	1.5%	236.35	4.7
1152	menthone	trans-(2S,5R)-5-methyl-2-propan-2-ylcyclohexan-1-one	14073-97-3	1.4%	154.25	2.7
1415	trans-beta- caryophyllene	(1R,4E,9S)-4,11,11-trimethyl-8-methylidenebicyclo[7.2.0]undec-4-ene	87-44-5	1.4%	204.35	4.4
1106	cis-rose oxide	4-methyl-2-(2-methylprop-1-enyl)oxane	876-17-5	1.4%	154.25	2.9
1380	beta-bourbonene	(1S,2R,6S,7R,8S)-1-methyl-5-methylidene-8-propan-2-yltricyclo[5.3.0.02,6]decane	5208-59-3	1.3%	204.35	4.7
1512	delta-cadinene	(1S,8aR)-4,7-dimethyl-1-propan-2-yl-1,2,3,5,6,8a-hexahydronaphthalene	483-76-1	1.1%	204.35	3.8
1576	phenyl ethyl tiglate	2-phenylethyl (E)-2-methylbut-2-enoate	55719-85-2	1.1%	204.26	3.3
1464	geranyl propionate	[(2E)-3,7-dimethylocta-2,6-dien-1-yl] propanoate	105-90-8	1.0%	210.31	3.9

**Table A5:** Constituent profile from GC–MS analysis of lavender oil.

RI	Compound	IUPAC	CAS	%AUC	MW (g/mol)	LogP
1099	linalool	3,7-dimethylocta-1,6-dien-3-ol	78-70-6	32.2%	154.25	2.7
1249	linalyl acetate	3,7-dimethylocta-1,6-dien-3-yl acetate	115-95-7	28.3%	196.29	3.3
1449	trans-beta- farnesene	(6E)-7,11-dimethyl-3-methylidenedodeca-1,6,10-triene	18794-84-8	5.0%	204.35	6.2
1280	lavandulyl acetate	(5-methyl-2-prop-1-en-2-ylhex-4-en-1-yl) acetate	25905-14-0	4.6%	196.29	3.6
1033	cis-beta-ocimene	(3Z)-3,7-dimethylocta-1,3,6-triene	3338-55-4	4.3%	136.23	4.3
1415	trans-beta- caryophyllene	(1R,4E,9S)-4,11,11-trimethyl-8-methylidenebicyclo[7.2.0]undec-4-ene	87-44-5	3.9%	204.35	4.4
1178	terpinen-4-ol	4-methyl-1-propan-2-ylcyclohex-3-en-1-ol	562-74-3	3.6%	154.25	2.2
1043	trans-beta-ocimene	(3E)-3,7-dimethylocta-1,3,6-triene	3779-61-1	2.9%	136.23	4.3
1192	alpha-terpineol	2-(4-methylcyclohex-3-en-1-yl)propan-2-ol	98-55-5	1.4%	154.25	1.8
981	3-octanone	Octan-3-one	106-68-3	1.1%	128.21	2.3
1029	1,8-cineole	1,3,3-trimethyl-2-oxabicyclo[2.2.2]octane	470-82-6	1.0%	154.25	2.5

**Table A6:** Constituent profile from GC–MS analysis of lemongrass oil.

RI	Compound	IUPAC	CAS	%AUC	MW (g/mol)	LogP
1270	geranial	(2E)-3,7-dimethylocta-2,6-dienal	141-27-5	41.5%	152.23	3.0
1240	neral	(2Z)-3,7-dimethylocta-2,6-dienal	106-26-3	31.1%	152.23	3.0
1250	geraniol	(2E)-3,7-dimethylocta-2,6-dien-1-ol	106-24-1	7.5%	154.25	2.9
1375	geranyl acetate	(2E)-3,7-dimethylocta-2,6-dien-1-yl acetate	105-87-3	5.0%	196.29	3.5
1177	trans-isocitral	(3E)-3,7-dimethylocta-3,6-dienal	72203-98-6	1.5%	152.23	2.9
1415	trans-beta- caryophyllene	(1R,4E,9S)-4,11,11-trimethyl-8-methylidenebicyclo[7.2.0]undec-4-ene	87-44-5	1.3%	204.35	4.4
1068	4-nonanone	Nonan-4-one	4485-09-0	1.2%	142.24	2.8
1099	linalool	3,7-dimethylocta-1,6-dien-3-ol	78-70-6	1.1%	154.25	2.7
981	6-methyl-5- hepten-2-one	6-methylhept-5-en-2-one	110-93-0	1.1%	126.2	1.9
1509	gamma-cadinene	(1R,4aS,8aS)-7-methyl-4-methylidene-1-propan-2-yl-2,3,4a,5,6,8a-hexahydro-1H-naphthalene	1460-97-5	1.1%	204.35	4.3

**Table A7:** Constituent profile from GC–MS analysis of spearmint oil.

RI	Compound	IUPAC	CAS	%AUC	MW (g/mol)	LogP
1249	carvone	(5R)-2-methyl-5-prop-1-en-2-ylcyclohex-2-en-1-one	6485-40-1	59.4%	150.22	2.4
1026	limonene	(4R)-1-methyl-4-prop-1-en-2-ylcyclohexene	5989-27-5	22.5%	136.23	3.4
1029	1,8-cineole	1,3,3-trimethyl-2-oxabicyclo[2.2.2]octane	470-82-6	2.0%	154.25	2.5
987	myrcene	7-methyl-3-methylideneocta-1,6-diene	123-35-3	1.7%	136.23	4.3
1380	beta-bourbonene	(1S,2R,6S,7R,8S)-1-methyl-5-methylidene-8-propan-2-yltricyclo[5.3.0.02,6]decane	5208-59-3	1.4%	204.35	4.7
1196	cis-dihydro-carvone	cis-(2R,5S)-2-methyl-5-prop-1-en-2-ylcyclohexan-1-one	3792-53-8	1.4%	152.23	2.7
975	beta-pinene	6,6-dimethyl-2-methylidenebicyclo[3.1.1]heptane	127-91-3	1.1%	136.23	3.1
1415	trans-beta- caryophyllene	(1R,4E,9S)-4,11,11-trimethyl-8-methylidenebicyclo[7.2.0]undec-4-ene	87-44-5	1.0%	204.35	4.4

**Table A8:** Constituent profile from GC–MS analysis of tea tree oil.

RI	Compound	IUPAC	CAS	%AUC	MW (g/mol)	LogP
1178	terpinen-4-ol	4-methyl-1-propan-2-ylcyclohex-3-en-1-ol	562-74-3	41.4%	154.25	2.2
1052	gamma-terpinene	1-methyl-4-propan-2-ylcyclohexa-1,4-diene	99-85-4	20.6%	136.23	2.8
1010	alpha-terpinene	1-methyl-4-propan-2-ylcyclohexa-1,3-diene	99-86-5	10.7%	136.23	2.8
1029	1,8-cineole	1,3,3-trimethyl-2-oxabicyclo[2.2.2]octane	470-82-6	3.8%	154.25	2.5
1085	terpinolene	1-methyl-4-propan-2-ylidenecyclohexene	586-62-9	3.5%	136.23	2.8
1187	alpha-terpineol	2-(4-methylcyclohex-3-en-1-yl)propan-2-ol	98-55-5	3.0%	154.25	1.8
925	alpha-pinene	2,6,6-trimethylbicyclo[3.1.1]hept-2-ene	80-56-8	2.5%	136.23	2.8
1021	para-cymene	1-methyl-4-propan-2-ylbenzene	99-87-6	1.9%	134.22	4.1
918	alpha-thujene	2-methyl-5-propan-2-ylbicyclo[3.1.0]hex-2-ene	2867-05-2	1.0%	136.23	2.8
1026	limonene	(4R)-1-methyl-4-prop-1-en-2-ylcyclohexene	5989-27-5	1.0%	136.23	3.4

## Appendix B

Historical data from LLNA studies performed on essential oils derived from the same botanical source as the studied materials are shown below in Table A9. EC3 values are provided as the percentage of the test material in conjunction with their corresponding NESIL values and reference citations.

**Table A9:** Summary of historical LLNA data for the evaluated materials.

Material Name	EC3 (%)	NESIL_LLNA_ (µg/cm^2^)	Reference
Cedarwood Oil	22%	5500	[33]
Cinnamon Bark Oil	n/a	n/a	n/a
Clove Bud Oil	7.1%	1775	[34]
Geranium Oil	>50%	>12,500	[35]
Lavender Oil	36%	9000	[36]
Lemongrass Oil	6.5%	1625	[35]
Spearmint Oil	8.2%	2050	[34]
Tea Tree Oil	4.4%	1100	[37]
Tea Tree Oil	8.3%	2075	[37]
Tea Tree Oil	24.3%	6075	[37]
Tea Tree Oil	25.5%	6375	[37]

n/a, not available/applicable.
