# Supplementary material for: Comparative Analysis of Test Methods for Predicting Dermal Sensitization Potency in Essential Oils: A Component-Based Prediction Model, GARDskin Dose–Response Assay, and Local Lymph Node Assay
Source: Toxics. 2026 Sep 9;14(9):800. doi: 10.3390/toxics14090800 (PMC13611667; doi:10.3390/toxics14090800)
Supplement: Supplementary file 1 [file toxics-14-00800-s001.zip › toxics-4440210-supplementary.pdf]

Supplemental Materials for Dawson et al. "Comparative analysis of test methods for predicting dermal sensitization potency in essential oils: a component-based prediction model, GARDskin dose–response assay, and local lymph node assay."

Storage Conditions and Sampling: Following manufacturing, the test materials were stored at ambient room temperature away from sunlight in bulk drums and totes. Representative samples of each material lot were sampled using a standard technique and tested according to physical, microbiological, and analytical specifications, including GC–MS. Samples requisitioned for GARDskin DR were obtained directly from bulk lots and stored at ambient room temperature away from light in 5 mL amber glass bottles with a standard orifice reducer and cap until the time of testing.

Table S1. Manufacture, GC–MS, and GARDskin DR test dates for the examined materials.

Material	Manufacture Date	GC–MS Date	GARDskin DR Date
Cedarwood Oil	2025-08-12	2025-10-01	2026-01-27
Cinnamon Bark Oil	2024-06-01	2024-11-27	2025-06-03
Clove Bud Oil	2024-08-05	2024-11-26	2025-02-18
Geranium Oil	2024-10-08	2025-06-28	2026-01-28
Lavender Oil	2024-07-11	2024-12-13	2025-02-18
Lemongrass Oil	2025-01-08	2025-07-02	2026-01-28
Spearmint Oil	2023-07-24	2024-05-16	2026-01-28
Tea Tree Oil	2024-02-01	2024-10-22	2025-02-18

Table S2. Results of primary and sensitivity statistical comparisons between the CP, GARDskin DR, and LLNA outputs for the test materials.

Analysis	Primary Analysis (All Materials)	N	Sensitivity Analysis (Excluding Geranium)	N	Sensitivity Analysis (Excluding Tea Tree)	N
Spearman rho: CP-GARD	0.881, p = 0.007	8	0.929, p = 0.007	7	0.929, p = 0.007	7
Spearman rho: CP-LLNA	0.929, p = 0.007	7	0.886, p = 0.034	6	0.943, p = 0.017	6
Spearman rho: GARD-LLNA	0.607, p = 0.17	7	0.600, p = 0.24	6	0.771, p = 0.100	6
Bland Altman bias and 95% LOA: logCP-logGARD	0.35 (-0.22, 0.92)	8	0.30 (-0.24, 0.83)	7	0.42 (-0.01, 0.86)	7
Bland Altman bias and 95% LOA: logCP-logLLNA	0.49 (-0.06, 1.04)	7	0.45 (-0.11, 1.02)	6	0.43 (-0.08, 0.94)	6
Bland Altman bias and 95% LOA: logGARD-logLLNA	0.18 (-0.75, 1.12)	7	0.22 (-0.79, 1.23)	6	0.05 (-0.64, 0.74)	6
Friedman Test	$\chi^2(2) = 7.71$, p = 0.021	7	$\chi^2(2) = 6.33$, p = 0.042	6	$\chi^2(2) = 9.33$, p = 0.009	6
Wilcoxon Statistic and Holm Adjusted p-value: logCP-logGARD	W = 2.0, p = 0.094	7	W = 2.0, p = 0.188	6	W = 0.0, p = 0.094	6
Wilcoxon Statistic and Holm Adjusted p-value: logCP-logLLNA	W = 0.0, p = 0.047	7	W = 0.0, p = 0.094	6	W = 0.0, p = 0.094	6
Wilcoxon Statistic and Holm Adjusted p-value: logGARD-logLLNA	W = 10.0, p = 0.578	7	W = 6.0, p = 0.438	6	W = 10, p = 1.000	6
Kendall's W	0.857, p = 0.017	7	0.860, p = 0.024	6	0.911, p = 0.018	6

Figure S1. Cedarwood Oil GC–MS Chromatogram and Chemical Composition

Sample Information:
Analyzed by: Ambika Poudel
Analyzed: 10/1/2025 2:10:03 PM
Sample Type: Essential Oil

R. Time	Name	Area%
12.709	alpha-Pinene	0.26
22.045	Terpinolene	0.01
36.596	gamma-Neoclovene	0.08
40.071	Guanidiene isomer	0.06
42.145	2-epi-alpha-Funebrene	0.85
42.355	alpha-Isocomene	0.08
42.429	alpha-Duprezianene	0.74
42.544	7-epi-Sesquithujene	0.21
42.61	beta-Elemene	0.03
42.853	iso-Longifolene	0.22
43.357	beta-Longipinene	0.2

Sample Name: Cedarwood Virginiana
Lot#: WSD250804C SAP# 275326
Injection Volume: 0.30

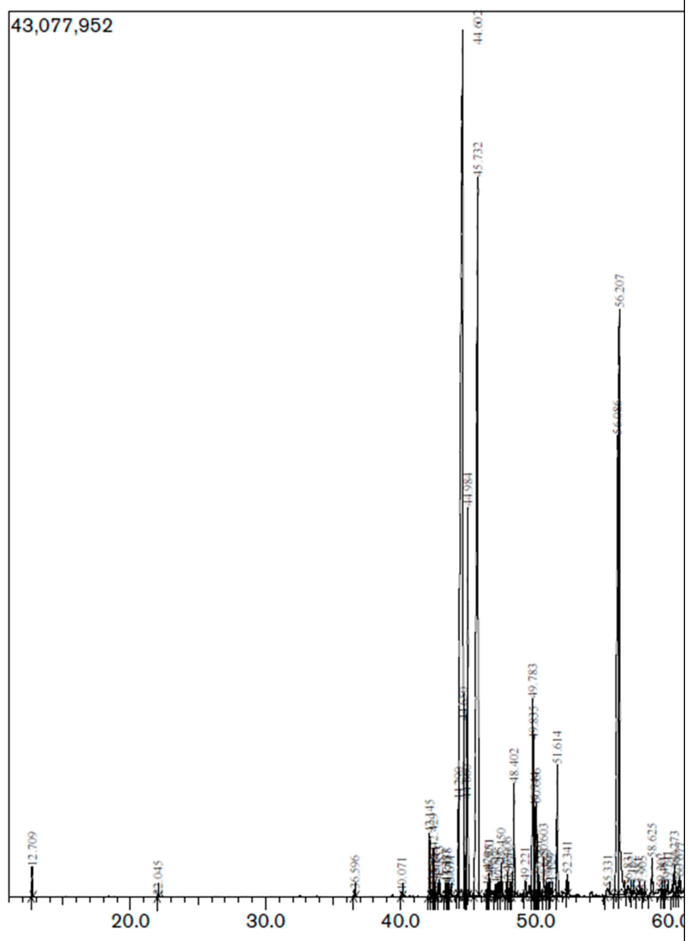

43.438	alpha-Funebrene	0.2
43.57	Sesquithujene	0.05
43.671	Di-epi-alpha-cedrene	0.13
44.29	beta-Funberene	0.59
44.602	alpha-Cedrene	31.8
44.65	beta-Caryophyllene	0.86
44.88	beta-Duprezianene	0.77
44.984	beta-Cedrene	5.81
45.732	cis-Thujopsene	19.63
46.42	beta-Barbatene	0.14
46.551	Prezizaene	0.25
46.57	trans-beta-Farnesene	0.18
47.047	allo-Aromadendrene	0.16
47.295	alpha-Acoradiene	0.18
47.45	beta-Acoradiene	0.42
47.906	10-epi-beta-Acoradiene	0.37
47.965	trans-Cadina-1(6),4-diene	0.09
48.17	gamma-Curcumene	0.18
48.402	gamma-Gurjunene	1.6
49.221	beta-Alaskene	0.19
49.783	alpha-Cuprenene	2.72
49.835	Pseudowiddrene	1.34
49.944	alpha-Chamigrene	1.07
50.056	Cuparene	1.29
50.201	alpha-Alaskene	0.28
50.255	1,2-Dihydro cuparene	0.1
50.603	Alaskene isomer	0.5
50.859	1,4-Dihydro cuparene	0.19
50.96	beta-Sesquiphellandrene	0.05
51.129	trans-gamma-Bisabolene	0.09
51.614	gamma-Cuprenene	1.77
52.341	delta-Cuprenene	0.27
55.331	Allo-cedrol	0.25
56.086	Widdrol	10.23
56.207	Cedrol	10.85
56.831	Epi-Cedrol	0.16
57.162	2-epi-alpha-Cedren-3-one	0.04
57.581	alpha-Acorenol	0.07
57.905	1,7-Di-epi-alpha-cedrenal	0.07
58.625	3-Thujopsanone	0.64
59.302	1,7-di-epi-beta-Cedrenal	0.1
59.481	beta-Bisabolol	0.07
59.741	Acorenone	0.16
60.273	epi-alpha-Bisabolol	0.46
60.36	alpha-Bisabolol	0.18
60.677	iso-Thujopsanone	0.25
61.82	trans-Thujopsenal	0.24
61.96	Mayurone isomer	0.09
66.515	Nootkatone	0.16

Figure S2. Cinnamon Bark Oil GC–MS Chromatogram and Chemical Composition

Sample Information:
Analyzed by: Ambika Poudel
Analyzed: 11/27/2024 10:33:18 PM
Sample Type: Essential Oil
Sample Name: Cinnamon Bark
Lot#: PTC240614A SAP# 267577
Injection Volume: 0.30

R. Time	Name	Area%
10.229	Styrene	0.05
11.703	alpha-Thujene	0.39
12.116	alpha-Pinene	1.96
12.912	alpha-Fenchene	0.02
13.01	Camphene	0.63
13.933	Benzaldehyde	0.26
14.261	Sabinene	0.11
14.561	beta-Pinene	0.49
15.172	Myrcene	0.13
16.266	alpha-Phellandrene	2.1
16.363	delta-3-Carene	0.15
16.895	alpha-Terpinene	0.86
17.093	meta-Cymene	0.04
17.443	para-Cymene	2.35
17.689	Limonene	0.96
17.827	beta-Phellandrene	3.09
17.875	1,8-cineole	0.15
18.063	cis-beta-Ocimene	0.07
18.739	trans-beta-Ocimene	0.05
19.529	gamma-Terpinene	0.12
21.293	Terpinolene	0.17
21.742	para-Cymenene	0.07
22.365	Linalool	2.91
22.471	2-Methyl butyl-2-methyl butyrate	0.06
26.84	Benzenepropanal	0.21
27.312	Isoborneol	0.03
27.495	Borneol	0.02
27.891	Terpinen-4-ol	0.18
28.948	alpha-Terpineol	0.34
29.502	cis-Sabinol	0.02

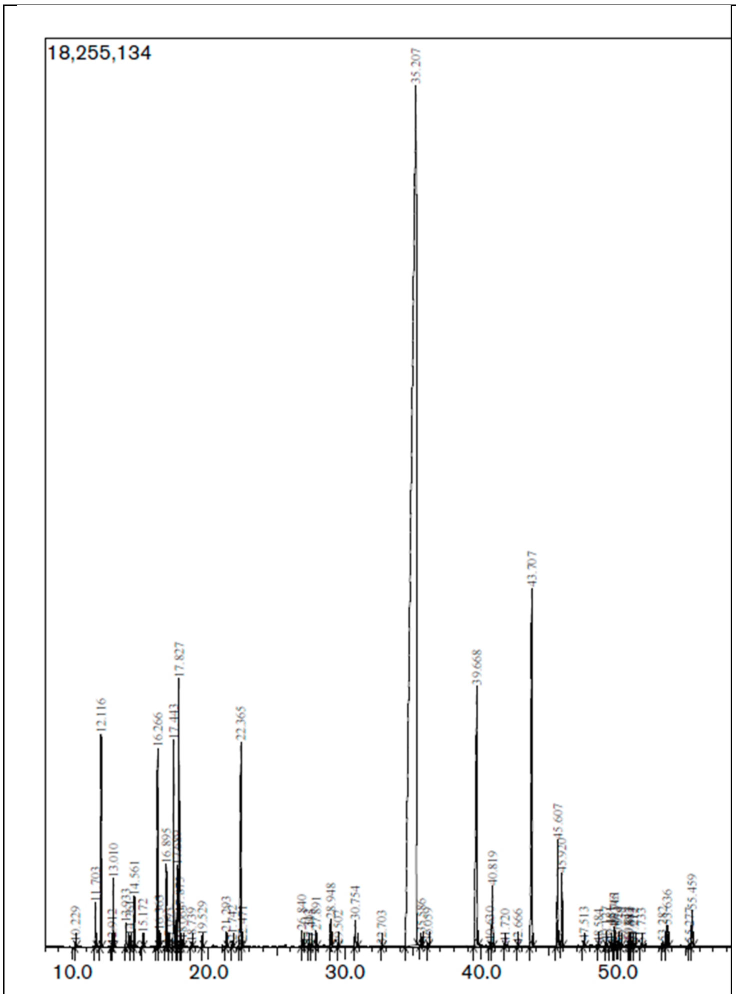

30.754	cis-Cinnamaldehyde	0.39
32.703	Carvotanacetone	0.02
35.207	trans-Cinnamaldehyde	64.17
35.586	Safrole	0.15
36.059	Carvacrol	0.04
39.668	Eugenol	5.36
40.63	Hydro cinnamyl acetate	0.05
40.819	alpha-Copaene	0.8
41.72	beta-Elemene	0.03
42.666	cis-Caryophyllene	0.02
43.707	beta-Caryophyllene	5.84
45.607	trans-Cinnamyl acetate	1.52
45.92	alpha-Humulene	0.96
47.513	ar-Curcumene	0.07
48.584	alpha-Muurolene	0.03
49.127	beta-Bisabolene	0.05
49.481	Tridecanal+gamma-Cadinene	0.06
49.745	delta-Cadinene	0.08
49.761	Eugenol acetate	0.19
50.024	trans-Calamenene	0.05
50.238	trans-gamma-Bisabolene	0.03
50.813	trans-ortho-Methoxy cinnamaldehyde	0.09
50.882	alpha-Cadinene	0.06
51.073	trans-alpha-Bisabolene	0.06
51.212	alpha-Calacorene	0.04
51.733	Isocaryophyllene oxide	0.05
53.282	Caryophyllene alcohol	0.1
53.636	Caryophyllene oxide	0.32
55.277	Humulene epoxide II	0.04
55.459	Tetradecanal	0.51

Figure S3. Clove Bud Oil GC–MS Chromatogram and Chemical Composition

Sample Information:
Analyzed by: Ambika Poudel
Analyzed: 11/26/2024 8:44:25 PM
Sample Type: Essential Oil
Sample Name: Clove Bud
Lot#: IND240816A SAP# 267678
Injection Volume: 0.30

R. Time	Name	Area%
8.119	Furfural	0.05
10.437	2-Heptanone	0.01
10.991	2-Heptanol	0
14.22	5-Methyl furfural	0
14.241	Benzaldehyde	0.01
15.55	6-Methyl-5-hepten-2-one	0
16.353	Ethyl hexanoate	0
18.272	Limonene	0
18.839	2-Heptyl acetate	0.02
19.353	trans-beta-Ocimene	0
22.304	2-Nonanone	0.01
22.58	Methyl benzoate	0
22.894	Linalool	0.01
23.802	2-Methyl-6-methylen-octa-1,7-dien-3-one	0.01
27.172	Benzyl acetate	0.01
27.718	Ethyl benzoate	0.01
29.184	Methyl salicylate	0.15
33.253	Chavicol	0.09
40.644	Eugenol	72.98
41.149	neo-Caryophyllene	0.05
41.593	alpha-Copaene	0.12
42.457	beta-Elemene	0.01
43.127	Methyl eugenol	0.03
43.403	cis-Caryophyllene	0.03
43.789	Isocaryophyllene	0.01
44.445	beta-Caryophyllene	8.73
44.869	Unidentified	0.04
46.198	trans-Muurola-3,5-diene	0.06
46.485	Unidentified	0.03
46.589	alpha-Humulene	1.37
46.843	allo-Aromadendrene	0.01
47.596	cis-Cadina-1(6),4-diene	0.02
47.777	trans-Cadina-1(6),4-diene	0.02
48.039	alpha-Amorphene	0.01
48.644	beta-Selinene	0.01

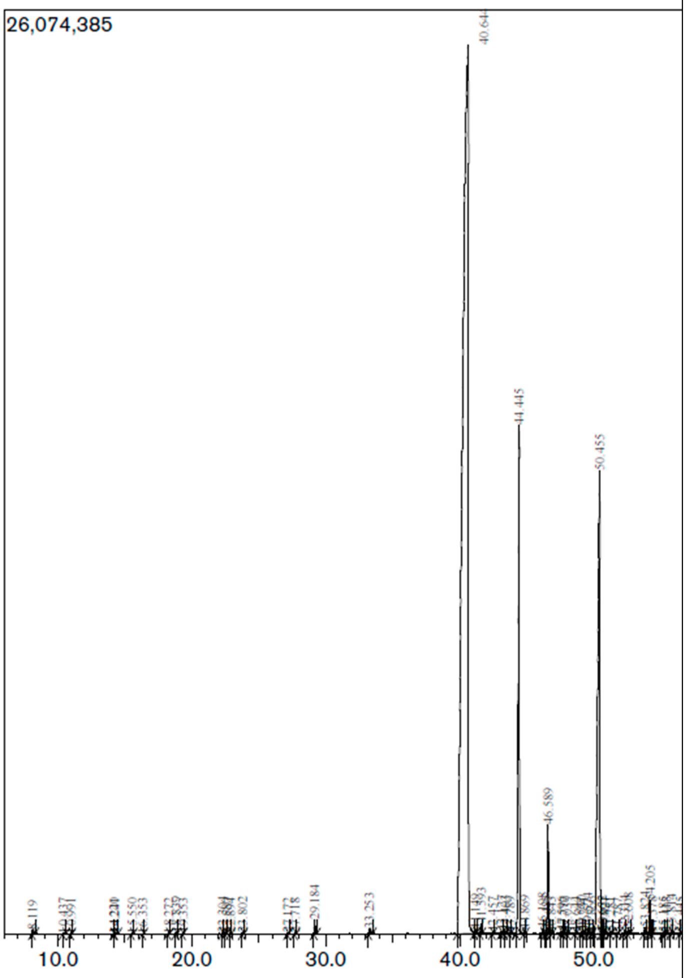

Figure S4. Geranium Oil GC–MS Chromatogram and Chemical Composition

Sample Information:
Analyzed by: Ambika Poudel
Analyzed: 6/28/2025 8:00:48 AM
Sample Type: Essential Oil
Sample Name: Geranium
Lot#: FK241016I SAP# 270836
Injection Volume: 0.30

R. Time	Name	Area%
12.693	alpha-Pinene	0.49
15.827	Myrcene	0.08
18.354	Limonene	0.14
21.058	cis-Linalool oxide (furanoid)	0.23
22.125	trans-Linalool oxide (furanoid)	0.1
23.093	Linalool	4.37
23.747	cis-Rose oxide	1.37
24.843	trans-Rose oxide	0.58
26.628	Citronellal	0.16
26.921	Menthone	1.43
27.609	Isomenthone	6.3
29.626	alpha-Terpineol	0.23
32.155	Citronellol	32.1
32.627	Neral	0.44
33.715	Geraniol	11.75
34.638	Geranial	0.75
35.066	Citronellyl formate	10.03
36.67	Geranyl formate	3.62
39.874	alpha-Cubebene	0.27
40.018	Citronellyl acetate	0.41
41.757	alpha-Copaene	0.63
41.876	Geranyl acetate	0.57
42.289	beta-Bourbonene	1.33
44.5	beta-Ylangene	0.15
44.574	beta-Caryophyllene	1.42
45.197	beta-Copaene	0.15
45.811	Citronellyl propionate	0.57
45.919	6,9-Guaiadiene	0.33
46.407	Isogermacrene D	0.59
46.807	alpha-Humulene	0.33
47.079	allo-Aromadendrene	0.22
47.634	Geranyl propionate	0.98
47.834	trans-Cadina-1(6),4-diene	0.28
48.412	Germacrene D	1.64
49.09	alpha-Selinene	0.04
49.231	alpha-Muurolene	0.02
49.574	trans-trans-alpha-Farnesene	0.06
49.866	Unidentified	0.01
50.455	Eugenol acetate	15.08
50.622	trans-Calamenene	0.04
50.665	Zonarene	0.02
50.821	Unidentified	0.03
51.281	trans-Cadine-1,4-diene	0.02
51.787	alpha-Calacorene	0.01
52.314	Isocaryophyllene oxide	0.04
52.608	(2R-5-trans)-Caryophyll-5-en-12-al	0.08
53.824	Caryophyllene alcohol	0.09
54.205	Caryophyllene oxide	0.42
54.378	Isocaryophyllene alcohol	0.01
55.185	cis-Bisabol-11-ol	0.01
55.418	Sesquiterpeneol	0.01
55.814	Humulene epoxide II	0.04
56.445	Widdrol isomer	0.01
56.83	1-epi-Cubenol	0.01
57.054	Unidentified	0.01
57.493	Unidentified	0.01
59.982	2',3',4'-Trimethoxy acetophenone	0.01
62.377	Coniferyl aldehyde	0.01
64.421	Benzyl benzoate	0.01

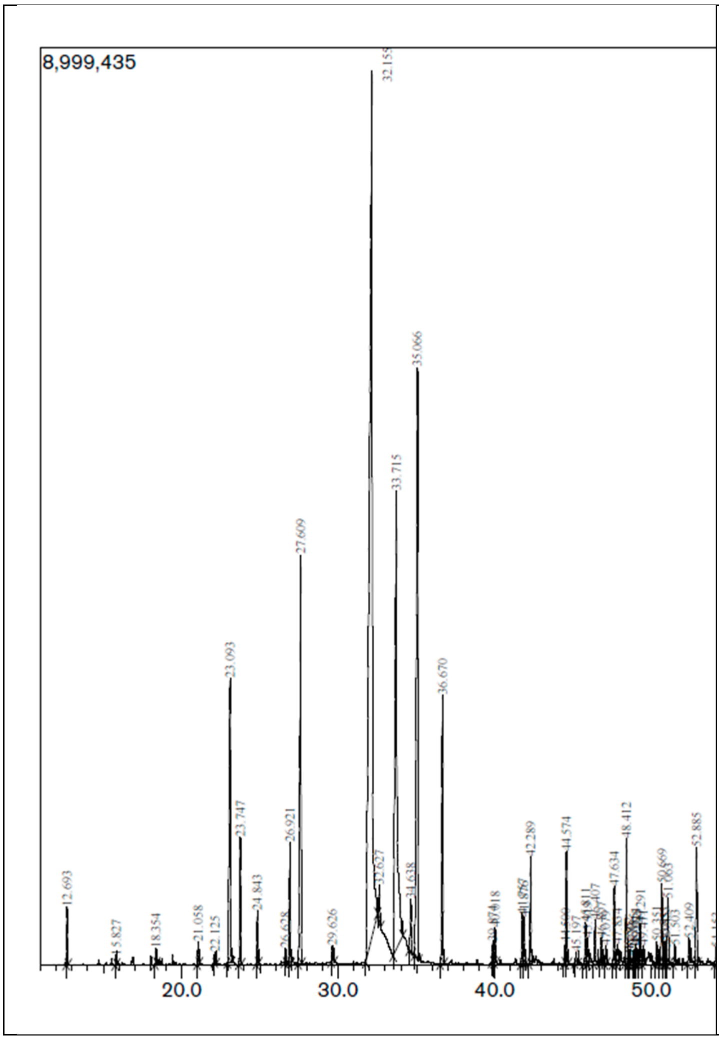

48.562	Selinadiene	0.13
48.595	Phenyl ethyl 2-methyl butanoate	0.05
48.885	beta-Selinene	0.19
49.014	Viridiflorene	0.21
49.025	trans-Muurola-4(14),5-diene	0.19
49.291	Bicyclogermacrene	0.59
49.471	alpha-Muurolene	0.24
50.351	gamma-Cadinene	0.28
50.669	delta-Cadinene	1.1
50.853	trans-Calamenene	0.3
50.945	Zonarene	0.17
51.063	Citronellyl butanoate	0.82
51.503	trans-Cadina-1,4-diene	0.24
52.409	alpha-Agarofuran	0.32
52.885	Geranyl butanoate	1.5
54.153	Spathulenol	0.31
54.467	Phenyl ethyl tiglate	1.08
56.675	Citronellyl valerate	0.16
56.861	10-epi-gamma-Eudesmol	4.96
57.084	1-epi-Cubenol	0.18
58.435	Geranyl valerate	0.23
58.61	Valerianol	0.27
58.621	alpha-Eudesmol	0.32
58.975	Citronellyl tiglate	0.46
60.103	Citronellyl isohexanoate	0.14
60.884	Geranyl tiglate	1.5

Figure S5. Lavender Oil GC–MS Chromatogram and Chemical Composition

Sample Information:
Analyzed by: Ambika Poudel
Analyzed: 12/13/2024 1:41:10 AM
Sample Type: Essential Oil
Sample Name: Lavender
Lot#: 503-06.08.2024 SAP# 267801
Injection Volume: 0.30

R. Time	Name	Area%
7.818	1-Methoxy hexane	0.12
9.453	n-Hexanol	0.05
12.177	alpha-Thujene	0.09
12.583	alpha-Pinene	0.19
13.483	Camphene	0.17
14.754	Sabinene	0.05
15.052	beta-Pinene	0.04
15.121	1-Octen-3-ol	0.1
15.44	3-Octanone	1.05
15.688	Myrcene	0.86
16.041	Butyl butanoate	0.12
16.137	3-Octanol	0.13
16.744	alpha-Phellandrene	0.04
16.894	delta-3-Carene	0.11
17.055	Hexyl acetate	0.79
17.406	alpha-Terpinene	0.07
17.567	meta-Cymene	0.04
17.889	para-Cymene	0.14
18.194	Limonene	0.5
18.29	beta-Phellandrene	0.49
18.365	1,8-cineole	0.97
18.623	cis-beta-Ocimene	4.25
19.291	trans-beta-Ocimene	2.9
20.05	gamma-Terpinene	0.19
20.866	cis-Linalool oxide (furanoid)	0.13
21.853	Terpinolene	0.14
21.945	trans-Linalool oxide (furanoid)	0.07
23.138	Linalool	32.21
23.22	Hexyl propionate+Hotrienol	0.15
23.383	1-Octen-3-yl acetate	0.76
24.172	3-Octanol acetate	0.13
24.76	allo-Ocimene	0.16
26.067	Hexyl isobutyrate+Camphor	0.26
27.243	Lavandulol	0.92
27.802	Borneol	0.45

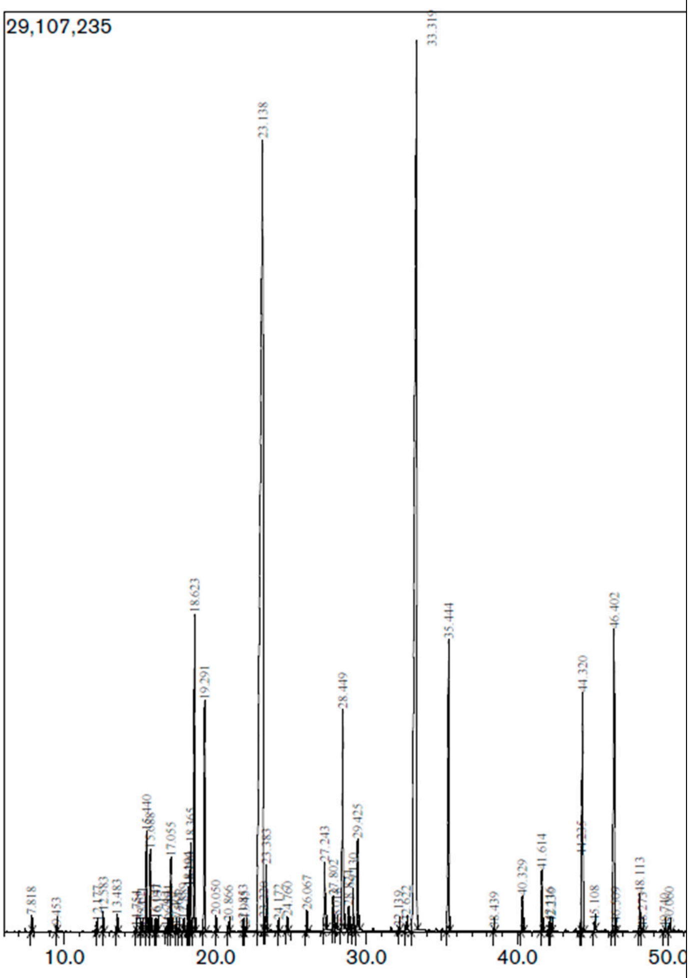

28.018	3-trans-5-cis-1,3,5-Undecatriene	0.08
28.449	Terpinen-4-ol	3.62
28.824	Cryptone	0.3
29.13	Hexyl butanoate	0.56
29.425	alpha-Terpineol	1.37
32.139	Hexyl 2-methyl butanoate	0.04
32.622	Cumin aldehyde	0.1
33.319	Linalyl acetate	28.3
35.444	Lavandulyl acetate	4.59
38.439	Hexyl tiglate	0.03
40.329	Neryl acetate	0.48
41.614	Geranyl acetate	0.79
42.116	Hexyl hexanoate	0.11
42.23	7-epi-Sesquithujene	0.11
44.235	alpha-Santalene	0.46
44.32	beta-Caryophyllene	3.92
45.108	trans-alpha-Bergamotene	0.11
46.402	trans-beta-Farnesene	5.04
46.509	alpha-Humulene	0.11
48.113	Germacrene D	0.55
48.273	trans-beta-Bergamotene	0.05
49.74	beta-Bisabolene	0.04
50.06	gamma-Cadinene	0.13
54.131	Caryophyllene oxide	0.21
57.587	epi-alpha-Cadinol	0.07

Figure S6. Lemongrass Oil GC–MS Chromatogram and Chemical Composition

Sample Information:
Analyzed by: Ambika Poudel
Analyzed: 7/2/2025 9:15:23 PM
Sample Type: Essential Oil
Sample Name: Lemongrass
Lot#: GA250113A SAP# 271582-1
Injection Volume: 0.30

R. Time	Name	Area%
12.515	Tricyclene	0.1
13.089	alpha-Pinene	0.16
14.033	Camphene	0.9
15.933	6-Methyl-5-hepten-2-one	1.09
16.253	Myrcene	0.06
16.35	Dehydro-1,8-Cineole	0.03
16.707	trans-Dehydroxy linalool oxide	0.04
17.105	n-Octanal	0.07
18.819	Limonene	0.23
18.93	beta-Phellandrene	0.02
19.203	cis-beta-Ocimene	0.28
19.899	trans-beta-Ocimene	0.17
21.565	4-Nonanone	1.24
22.531	Terpinolene	0.03
22.833	4-Nonanol	0.02
22.928	Rosefuran	0.1
23.39	Perillene	0.05
23.5	Linalool	1.11
23.927	alpha-Pinene oxide	0.09
26.157	Epiphotocitral A	0.12
26.477	exo-Isocitral	0.12
26.878	trans-Chrysanthemal	0.25
27.107	Citronellal	0.22
27.751	cis-Isocitral	0.94
28.301	Rosefuran epoxide	0.16
28.541	Borneol	0.14
29.072	trans-Isocitral	1.46
30.137	alpha-Terpineol	0.13
30.886	n-Decanal	0.19
32.23	Nerol	0.11
33.399	Neral	31.12
34.151	Geraniol	7.48
34.205	Piperitone	0.13
35.493	Geranial	41.47
35.583	4-Undecanone	0.05

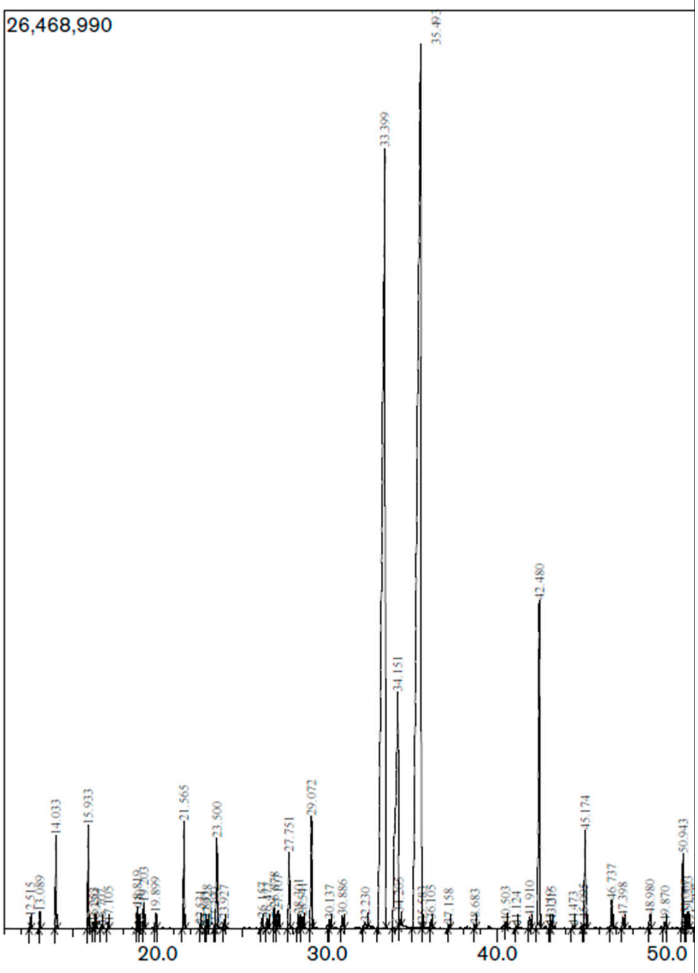

Figure S7. Spearmint Oil GC–MS Chromatogram and Chemical Composition

Sample Information:
Analyzed by: Ambika Poudel
Analyzed: 5/16/2024 11:40:39 AM
Sample Type: Essential Oil
Sample Name: Spearmint
Lot#: GA230803D SAP# 260288
Injection Volume: 0.30

R. Time	Name	Area%
11.09	2,5-Diethyl tetrahydrofuran	0.08
12.38	Hashishene	0.09
12.629	alpha-Thujene	0.04
13.071	alpha-Pinene	0.91
14.278	Methyl cyclohexanone	0.03
15.333	Sabinene	0.57
15.67	beta-Pinene	1.1
16.275	Myrcene	1.74
16.779	3-Octanol	0.2
17.235	para-Mentha-1(7),8-diene	0.08
17.307	3-ethenyl-1,2-dimethyl-1,4-Cyclohexadiene	0.04
18.06	alpha-Terpinene	0.13
18.601	para-Cymene	0.23
19.008	Limonene	22.5
19.081	beta-Phellandrene	0.07
19.174	1,8-cineole	1.97
19.23	cis-beta-Ocimene	0.06
19.94	trans-beta-Ocimene	0.07
20.778	gamma-Terpinene	0.22
21.669	cis-Sabinene hydrate	0.22
22.605	Terpinolene	0.1
23	para-Cymenene	0.04
23.63	Linalool	0.09
24.92	3-Octanol acetate	0.1
26.261	trans-Limonene oxide	0.07
27.641	Menthone	0.18
28.272	Isomenthone	0.06
28.668	delta-Terpineol	0.07
29.202	Menthol	0.54
29.36	Terpinen-4-ol	0.69
30.436	alpha-Terpineol	0.23
30.63	cis-Dihydro carvone	1.39
31.063	trans-Dihydro carvone	0.18
34.249	Carvone	59.44
34.351	Carvotanacetone	0.04
36.105	trans-Carvone oxide	0.09
37.158	Geranyl formate	0.04
38.683	Methyl geranate	0.04
40.503	Neric acid	0.05
41.124	Neryl acetate	0.02
41.91	alpha-Ylangene	0.2
42.48	Geranyl acetate	5.01
43.119	beta-Cubebene	0.03
43.215	beta-Elemene	0.09
44.473	Dodecanal	0.03
45.095	beta-Ylangene	0.06
45.174	beta-Caryophyllene	1.34
46.737	trans-Isoeugenol	0.38
47.398	alpha-Humulene	0.15
48.98	Germacrene D	0.19
49.87	epi-Cubebol	0.1
50.943	gamma-Cadinene	1.06
51.067	Cubebol	0.18
51.223	delta-Cadinene	0.23
51.718	trans-gamma-Bisabolene	0.14
52.323	alpha-Cadinene	0.03
52.993	alpha-Elemol	0.04
53.413	Geranyl butyrate	0.11
55.026	Caryophyllene oxide	0.48
56.634	Humulene epoxide II	0.04

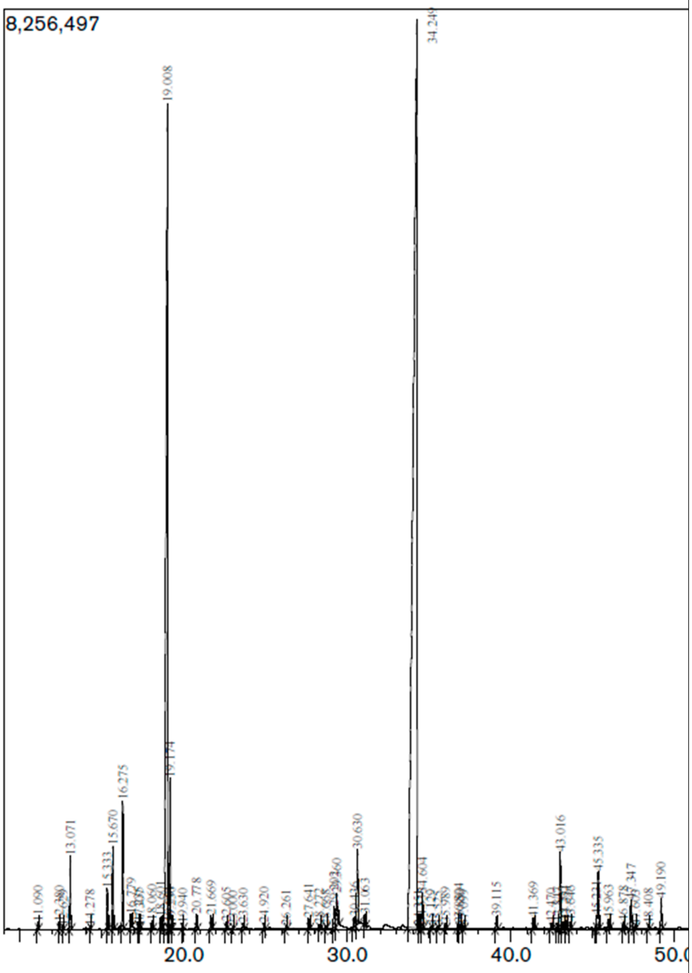

34.535	trans-Piperitone epoxide	0.02
34.604	Piperitone	0.56
35.129	cis-Carvone oxide	0.05
35.525	Isopiperitenone	0.04
35.989	trans-Carvone oxide	0.09
36.804	Menthyl acetate	0.18
36.85	Thymol	0.04
37.099	Dihydroedulan	0.04
39.115	Dihydro carvyl acetate	0.2
41.369	cis-Carvyl acetate	0.19
42.47	alpha-Copaene	0.11
42.847	Methyl propenyl hexahydro benzofuran	0.1
43.016	beta-Bourbonene	1.43
43.202	alpha-Bourbonene	0.12
43.351	beta-Elemene	0.15
43.646	cis-Jasmone	0.11
45.231	beta-Ylangene	0.22
45.335	beta-Caryophyllene	1.04
45.963	beta-Copaene	0.17
46.878	Isogermacrene D	0.13
47.347	trans-beta-Farnesene	0.56
47.603	alpha-Humulene	0.06
48.408	trans-Cadina-1(6),4-diene	0.09
49.19	Germacrene D	0.56
51.417	delta-Cadinene	0.06

Figure S8. Tea Tree Oil GC–MS Chromatogram and Chemical Composition

Sample Information:
Analyzed by: Ambika Poudel
Analyzed: 10/22/2024 1:51:22 AM
Sample Type: Essential Oil
Sample Name: Tea Tree
Lot#: FOKL240131C SAP# 263787
Injection Volume: 0.30

R. Time	Name	Area%
11.746	alpha-Thujene	0.97
12.164	alpha-Pinene	2.46
14.313	Sabinene	0.31
14.618	beta-Pinene	0.67
15.231	Myrcene	0.84
16.31	alpha-Phellandrene	0.39
17.042	alpha-Terpinene	10.69
17.509	para-Cymene	1.88
17.759	Limonene	0.97
17.915	beta-Phellandrene	0.92
17.97	1,8-cineole	3.79
19.772	gamma-Terpinene	20.6
21.424	Terpinolene	3.45
21.821	para-Cymenene	0.03
22.401	Linalool	0.05
22.53	trans-Sabinene hydrate	0.05
24.129	cis-para-Menth-2-en-1-ol	0.26
25.421	trans-para-Menth-2-en-1-ol	0.17
28.375	Terpinen-4-ol	41.42
29.168	alpha-Terpineol	2.99
38.01	delta-Elemene	0.04
39.011	alpha-Cubebene	0.04
40.909	alpha-Copaene	0.14
41.827	beta-Elemene	0.02
42.797	Methyl eugenol	0.02
42.915	beta-Maaliene	0.26
43.741	beta-Caryophyllene	0.3
44.211	gamma-Maaliene	0.05
44.654	alpha-Maaliene	0.05
44.957	Aromadendrene	0.78
45.02	Bourbon-11-ene	0.09
45.394	Selina-5,11-diene	0.08
45.415	cis-Muurola-3,5-diene	0.04
45.604	trans-Muurola-3,5-diene	0.09

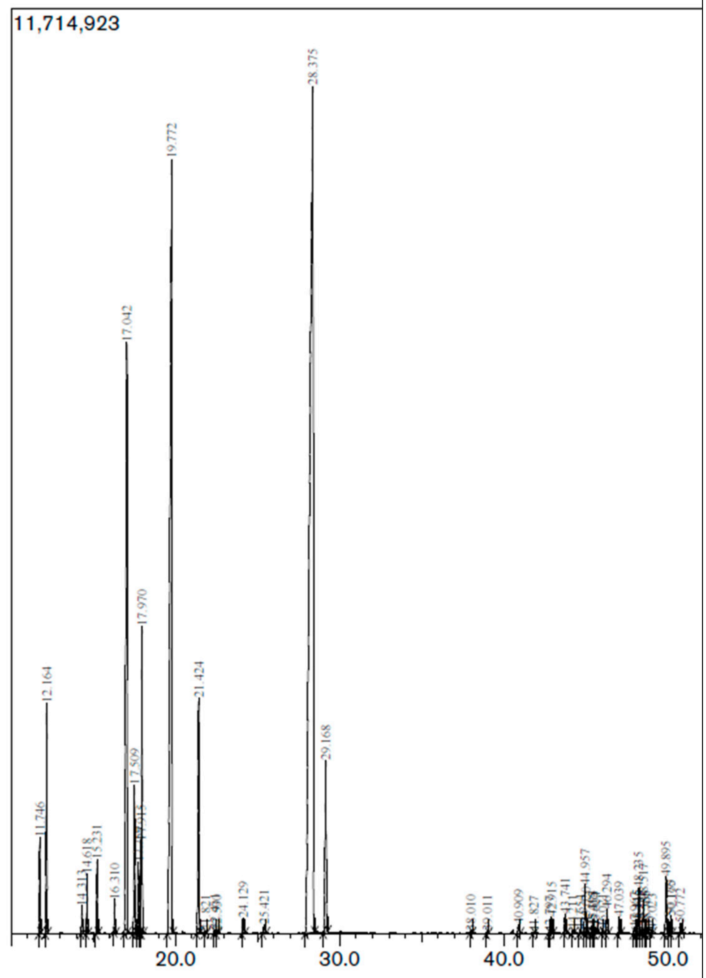

46.021	alpha-Humulene	0.06
46.294	allo-Aromadendrene	0.36
47.039	cis-Cadina-1(6),4-diene	0.28
47.967	delta-Selinene	0.09
48.115	beta-Selinene	0.14
48.235	Viridiflorene	0.72
48.295	trans-Muurola-4(14),5-diene	0.05
48.517	Bicyclogermacrene	0.56
48.585	alpha-Selinene	0.03
48.705	alpha-Muurolene	0.11
49.023	trans-trans-alpha-Farnesene	0.04
49.895	delta-Cadinene	0.89
50.165	trans-Calamenene	0.16
50.17	Zonarene	0.13
50.772	trans-Cadina-1,4-diene	0.15
52.589	Epiglobulol	0.06
53.035	Maaliol	0.04
53.052	Palustrol	0.04
53.483	Spathulenol	0.07
53.998	Globulol	0.31
54.503	Viridiflorol	0.15
54.635	Cubeban-11-ol	0.14
55.089	Ledol	0.04
55.226	Rosifoliol	0.07
56.199	Eudesmol isomer	0.14
56.397	1-epi-Cubenol	0.2
